# Innovative food supply chain through spatial computing technologies: A review

**DOI:** 10.1111/1541-4337.70055

**Published:** 2024-11-28

**Authors:** Peihua Ma, Xiaoxue Jia, Mairui Gao, Zicheng Yi, Shawn Tsai, Yiyang He, Dongyang Zhen, Ryan A. Blaustein, Qin Wang, Cheng‐I. Wei, Bei Fan, Fengzhong Wang

**Affiliations:** ^1^ Department of Nutrition and Food Science, College of Agriculture and Natural Resources University of Maryland College Park Maryland USA; ^2^ Beltsville Agricultural Research Center, Agriculture Research Service, United States Department of Agriculture Beltsville Maryland USA; ^3^ Department of Civil and Environmental Engineering, A. James Clark School of Engineering University of Maryland College Park Maryland USA; ^4^ Key Laboratory of Agro‐Products Processing Ministry of Agriculture and Rural Affairs, Chinese Academy of Agricultural Sciences Beijing China

**Keywords:** augmented reality, digital twin, food supply chain, spatial computing, virtual reality

## Abstract

The global food supply chain faces significant challenges related to inefficiencies, quality variability, and traceability issues, all of which contribute to food waste and consumer distrust. Spatial computing (SC) technologies, including augmented reality (AR), virtual reality (VR), and digital twins, offer promising solutions by enhancing precision agriculture, logistics, manufacturing, and retail operations. This review explores SC's potential across the entire food supply continuum, emphasizing improvements in resource management, supply chain transparency, and consumer engagement. Despite its promise, the widespread adoption of SC is limited by technical challenges and a lack of standardized protocols. The findings suggest that while SC has the potential to revolutionize the food supply chain by improving sustainability, efficiency, and safety, further interdisciplinary research and collaboration are essential to fully unlock its capabilities.

## 1 INTRODUCTION

The global food supply chain, a complex network extending from production to consumption, faces numerous challenges in the contemporary, rapidly evolving landscape (Davis et al., 2021; Lu et al., 2024). These challenges, including logistical inefficiencies, variances in quality control, and a lack of traceability, lead to considerable waste, undermine consumer confidence, and hinder the ability to respond effectively to market demands and food safety concerns (Godde et al., 2021). In this context, spatial computing (SC) emerges as a pioneering innovation, promising to fundamentally restructure the food supply chain. By integrating the physical and digital domains, SC facilitates the creation and interaction with digital objects within our tangible environments, enabling real‐time interactions and integrations that extend beyond traditional limitations (Çöltekin et al., 2020; Goldstein & Budiu, 2001). Through its capacity to enhance visibility, interactivity, and operational efficiency, SC offers a viable strategy for addressing the pressing challenges facing the food supply chain (Moshood et al., 2021). Encompassing a broad spectrum of technologies that merge digital elements with the physical world, SC enables interactions as if these digital entities coexisted physically (Ghazal & Alzoubi, 2021). Notably, SC technology has been recognized for its transformative potential at the highest levels of policy formulation, evidenced by its inclusion in the White House Critical and Emerging Technologies List for 2024. This recognition underscores the strategic importance of SC technologies in advancing national interests and addressing global challenges, further highlighting their relevance and potential impact within the food supply chain and beyond (Javed et al., 2022).

This review undertakes a comprehensive exploration of the application of SC within the food supply chain, aiming to elucidate how these technologies can overcome existing obstacles and enhance efficiency, sustainability, and safety along the food chain. Through an in‐depth examination of SC technologies, including augmented reality (AR) glasses, such as Meta's Ray‐Ban smart glasses and Xreal's Air 2 Ultra, virtual reality (VR) headsets like the Meta Quest 3 and Apple Vision Pro, as well as the critical digital twin (DT) technology—integral to Industry 4.0—this analysis reveals DT technology's role in creating virtual replicas of physical systems for advanced analysis, simulation, and control. This technology exemplifies the core principles of SC, showcasing its significant potential for operational optimization and efficiency enhancement. The application of SC within the food supply chain is explored through a comprehensive “farm to fork” perspective, addressing each critical juncture of the supply chain. Beginning with precision agriculture, this review summarizes recent reports of SC technologies that can revolutionize farming practices through enhanced resource management and optimized operational strategies. The discussion then transitions to transportation and logistics, where SC offers unparalleled opportunities for improving supply chain visibility and control, thereby enhancing the efficiency and reliability of food distribution. In the realm of smart manufacturing, the paper delves into the role of SC in streamlining food processing and packaging, emphasizing quality control and operational efficiency. Lastly, the consumer experience is examined, showcasing how SC can transform retail environments and personalize consumer–food interaction, enriching the purchasing and consumption experience.

**FIGURE 1:**
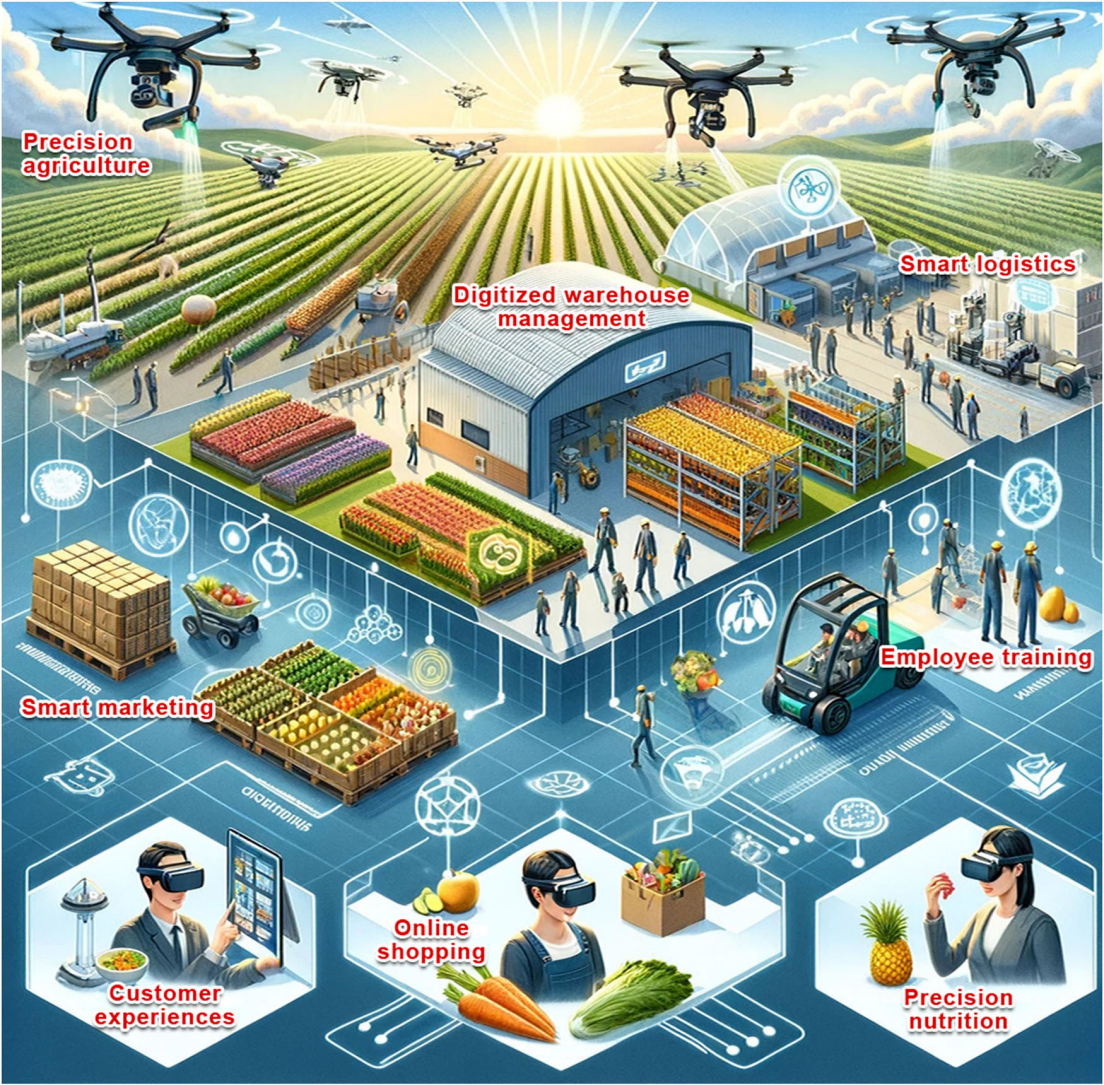
Illustration of spatial computing integration in the food supply chain.

Despite its promising applications, the widespread adoption of SC technologies in the food supply chain is not without challenges. Significant technical limitations remain, including the high costs of implementing SC infrastructure, the need for robust privacy and security protocols, and the absence of standardized industry frameworks. The development of clear regulatory guidelines and cross‐industry collaboration is essential to address these barriers and ensure the responsible integration of SC technologies across the food supply chain. This review undertakes a comprehensive exploration of the application of SC within the food supply chain, aiming to elucidate how these technologies can overcome existing obstacles and drive the future of food supply chain management. By presenting a structured analysis of SC technologies from farm to fork, this review highlights the transformative potential of SC in optimizing resource management, improving supply chain visibility, enhancing food manufacturing processes, and enriching consumer interactions. It further identifies key knowledge gaps and underscores the need for continued interdisciplinary research and collaboration to fully harness SC's capabilities in fostering a more sustainable, efficient, and secure global food system (Figure 1).

## 2 PRINCIPLE PERSPECTIVE

### 2.1 Data

**FIGURE 2:**
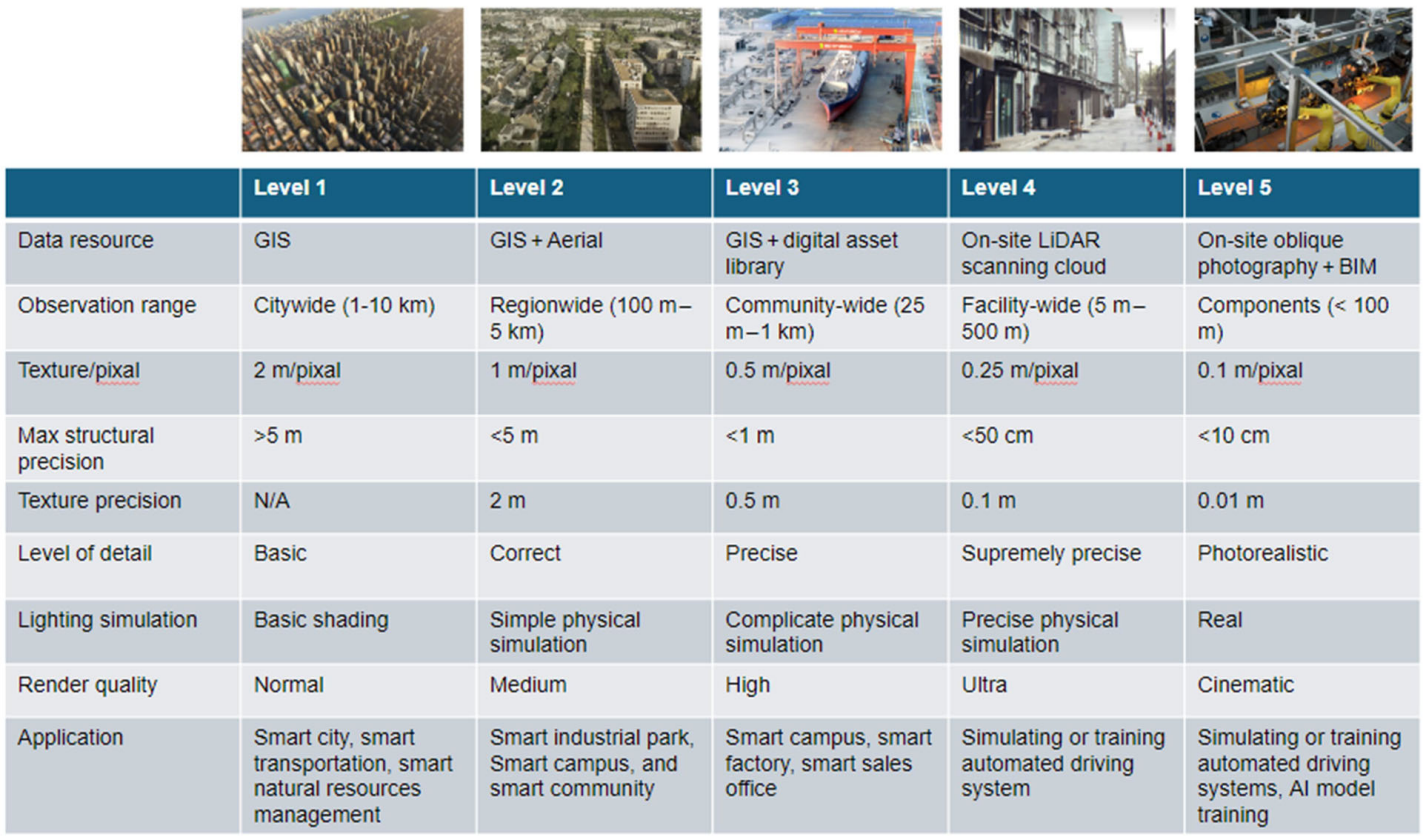
Summarize of spatial computing (SC) data precision level (L1–L5) and their applications. GIS, geographic information system; LiDAR, light detection and ranging.

In the domain of SC, the assortment and depth of data resources are instrumental in fostering innovation and enhancing efficiency within agriculture and the food sector (Sadeeq et al., 2021; Zhang & Zhang, 2022). These resources span a broad spectrum, including drone oblique photography data, light detection and ranging (LiDAR) scanning data, satellite vector data, OpenDrive/Building information model (BIM) data, digital assets libraries, photographic scanning models, and data generated through machine learning algorithms (White et al., 2021). This diversity in data types supports various quality levels of autogenerated landscapes and structures based on geographic information system (GIS) data, tailored to meet distinct needs ranging from large‐scale agricultural planning to detailed analysis for precision farming (Gill et al., 2022; Huang et al., 2021; Xu et al., 2021). The precision and application of SC data were summarized in Figure 2.

Level 1—Initial quality: At this foundational tier, data resources such as GIS and satellite photo offer basic precision suitable for extensive agricultural mapping and resource management. For example, with a maximum structural precision of 5 m and a broad observation range of 1–10 km, these resources are ideal for preliminary assessment of large agricultural areas, enabling the identification of broad patterns in crop distribution, land use, and resource allocation. Basic shading for lighting simulation suffices at this level of resolution, providing visual cues for topographical and vegetational analysis, thereby supporting strategic decisions in resource management and crop production planning.

Level 2—Medium quality: Progressing to medium quality, the integration of GIS data with satellite and drone imagery enhances the detail available for agricultural analysis, improving structural precision to less than 5 m and texture precision to 2 m. This refinement facilitates more granular studies of agricultural landscapes, including the assessment of crop health, irrigation planning, and the detection of pest infestations over larger farms or regions. The observation range, narrowed to 100 m from 5 km, along with correct levels of detail and simple physical lighting simulations, supports advanced precision in monitoring and managing large‐scale agricultural operations.

Level 3—High quality: High‐quality data resources utilize a blend of GIS data, satellite imagery, and digital asset libraries to support high‐precision applications within the food sector, especially for medium‐scale operational planning in food manufacturing and supply chain logistics. With observation ranges from 25 m to 1 km and improved texture precision to 0.5 m, these resources are ideal for detailed site selection for food processing plants, precision agriculture practices, and targeted supply chain interventions. Complex physical simulations for lighting enhance the accuracy of crop health monitoring, pest detection, and yield prediction, thereby supporting efficient resource management and production optimization.

Level 4—High simulation: At this level, on‐site LiDAR scanning data enable extremely precise spatial analyses, essential for designing and optimizing food manufacturing facilities and detailed supply chain modeling. The significant improvement in observation range (5 m–500 m) and precision (up to 50 cm for structural precision and 0.1 m for texture precision) facilitates the detailed mapping of indoor environments, critical for optimizing manufacturing processes, enhancing food safety protocols, and planning efficient logistics within confined spaces. Such detailed data are also crucial for simulating transportation routes within supply networks, ensuring minimal disruption and optimal delivery times.

Level 5—Full simulation: The highest quality of spatial data, based on models from on‐site oblique photography, offers unparalleled precision (up to 10 cm for structural and 10 cm for texture precision) within observation ranges less than 100 m. This level of detail is vital for photorealistic simulations of food production environments, enabling the virtual testing of manufacturing process changes without physical trial‐and‐error, thereby saving time and resources. Furthermore, it supports the most advanced applications in precision agriculture and supply chain logistics, such as millimeter‐precise monitoring of crop growth or the development of highly detailed supply chain models to minimize waste and enhance food security.

### 2.2 Software

SC leverages a myriad of sophisticated software technologies, each tailored to facilitate the creation, analysis, and integration of spatial data within computational contexts.

#### 2.2.1 3D graphics libraries and engines: Foundational pillars of visualization

Three‐dimensional (3D) graphics libraries such as OpenGL, DirectX, and Vulkan offer a fundamental layer for direct interaction with graphics processing units (GPUs), providing a comprehensive set of functions for rendering complex 3D scenes and effects. OpenGL, an open standard maintained by the Khronos Group, facilitates cross‐platform 3D graphics rendering (Shreiner, 2009), whereas DirectX, developed by Microsoft, is specifically designed for high‐performance applications on Windows platforms (J'lali, 2020). Vulkan, also from the Khronos Group, is heralded for its high‐efficiency and cross‐platform capabilities, enabling detailed control over GPU operations and memory management (Lapinski, 2017).

High‐level game engines like Unity and Unreal Engine abstract the complexities of direct GPU manipulation, offering robust environments for the development of 3D applications. Unity utilizes a scripting language based on C#, providing an extensive asset store and a user‐friendly interface, thereby enabling rapid development cycles (Hocking, 2022). Unreal Engine, with its advanced rendering capabilities and Blueprint Visual Scripting system, allows for the creation of visually stunning and complex interactive environments without extensive programming knowledge (Venter & Ogterop, 2022). These engines are instrumental not only in game development but also in simulations, architectural visualizations, and immersive VR experiences, owing to their sophisticated physics engines and material systems.

#### 2.2.2 Computer vision and image processing libraries: Interpreting the visual world

Computer vision and image processing libraries, notably OpenCV and Point Cloud Library (PCL), are critical for the analysis and interpretation of visual information. OpenCV offers a wide array of algorithms for real‐time image processing, feature detection, and object recognition, facilitating applications from facial recognition to autonomous vehicle navigation (Bradski, 2000). PCL specializes in the processing of point clouds—sets of data points in space—often generated by 3D scanners or stereo cameras, providing tools for filtering, feature extraction, and 3D model reconstruction (Rusu & Cousins, 2011). These libraries are essential for tasks requiring the conversion of two‐dimensional (2D) images to 3D representations, enabling applications in AR, robotics, and beyond.

#### 2.2.3 Machine learning and AI frameworks: Enabling intelligent spatial computing

Machine learning and artificial intelligence (AI) frameworks, such as TensorFlow, PyTorch, and Keras, underpin the development of algorithms capable of interpreting complex spatial data. TensorFlow, developed by Google, offers a versatile architecture for deploying computation across various platforms, from servers to edge devices, making it suitable for large‐scale and real‐time applications (Abadi et al., 2016). PyTorch, known for its dynamic computation graph and user‐friendly interface, facilitates rapid prototyping and research in AI. Keras, an application programming interface (API) designed for human beings rather than machines, operates atop TensorFlow, simplifying the creation and training of deep learning models (Paszke et al., 2019). These frameworks are integral to advancing SC applications, from scene understanding and object detection to generating 3D models from 2D images through convolutional neural networks (CNNs).

#### 2.2.4 Augmented and virtual reality SDKs: Crafting immersive experiences

Software development kits (SDKs) for AR and VR, such as ARCore, ARKit, Vuforia, and Oculus SDK, provide developers with comprehensive tools for creating immersive experiences (Linowes & Babilinski, 2017). ARCore and ARKit, developed by Google and Apple, respectively, offer features like motion tracking, environmental understanding, and light estimation for AR applications on mobile devices (Oufqir et al., 2020). Vuforia extends AR development capabilities with image recognition and supports a wide range of platforms, including Android, iOS, and Universial Windows Platform (UWP) (Simonetti Ibañez & Paredes Figueras, 2013). Oculus SDK focuses on VR, offering advanced features for head tracking, 3D audio, and hand presence for Oculus VR headsets (Desai et al., 2014). These SDKs are crucial for the development of applications that blend digital content with the physical world or create entirely virtual spaces, pushing the boundaries of human–computer interaction.

#### 2.2.5 Spatial data management and visualization tools: Understanding and representing spatial information

Tools and libraries for managing and visualizing spatial data, such as QGIS and Three.js, are indispensable for numerous SC applications. QGIS is an open‐source GIS software, providing extensive tools for spatial data analysis and visualization. It supports a wide range of raster and vector data formats, facilitating the examination and representation of geographic information. Three.js, a JavaScript library, enables the creation of animated and interactive 3D graphics within web browsers, without the need for plugins (Dirksen, 2013). It abstracts the complexities of WebGL, allowing for the straightforward integration of 3D content into web applications. These technologies are critical for visualizing spatial data in real time, enhancing the ability to interpret complex spatial relationships and phenomena.

#### 2.2.6 Sensor fusion frameworks: Integrating multimodal sensor data

Sensor fusion frameworks, such as the Robot Operating System (ROS), are essential for integrating data from diverse sensors like cameras, LiDAR, inertial measurement units (IMU), and global positioning systems (GPS; Macenski et al., 2022). Key strategies for data integration include Kalman filtering, which reduces noise for real‐time applications, and deep learning‐based fusion, which identifies patterns across data streams for improved accuracy.

ROS enables seamless synchronization of sensor inputs, allowing systems to accurately navigate and interpret complex environments. By fusing data from multiple sensors, SC systems can compensate for the limitations of individual sensors—for example, combining LiDAR's precise distance measurements with the rich visual data from cameras. This improves real‐time decision making, spatial awareness, and object detection, which is especially useful in autonomous navigation and industrial applications.

The benefits of sensor fusion have been demonstrated in enhanced precision and reliability in mapping and environmental monitoring. As these techniques advance, sensor fusion will be instrumental in expanding the capabilities of SC, transforming interactions between digital and physical spaces, and enabling more accurate, real‐time insights.

### 2.3 Hardware

**FIGURE 3:**
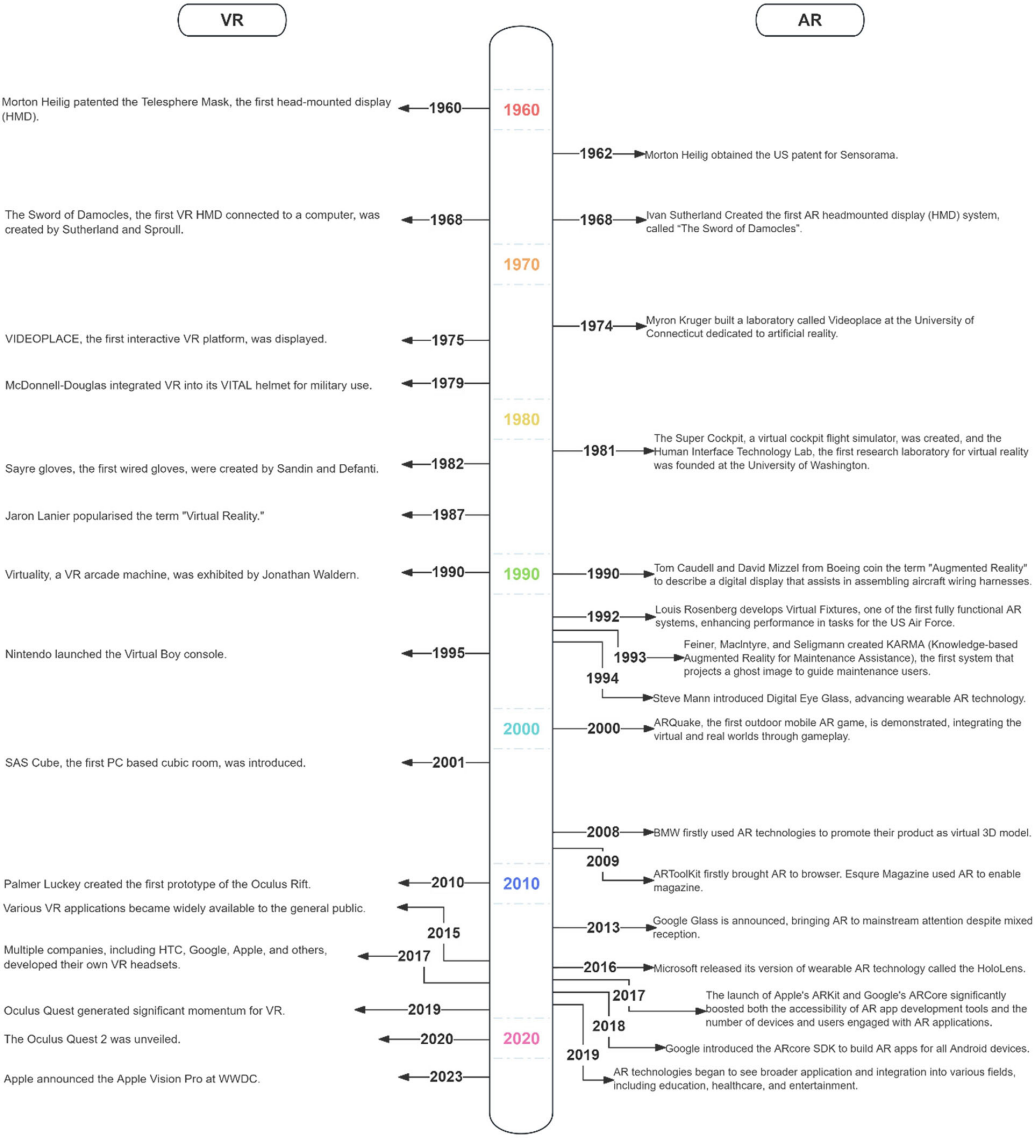
The development of spatial computing (SC) technology, virtual reality (VR) (left) and augmented reality (AR) (right) were summarized separately.

The hardware underpinning SC devices is a cornerstone of their functionality, enabling a myriad of applications. Typically, SC's hardware devices tend to be categorized into VR, AR, and mixed reality (MR) systems, but here we are going to take a more in‐depth look at dismantling SC's hardware (historical perspective of AR and VR was summarized in Figure 3). First, a critical component of these systems is the implementation of simultaneous localization and mapping (SLAM) technology, which is indispensable for real‐time environment mapping and device localization within it (Bresson et al., 2017; Debeunne & Vivet, 2020). SLAM technology leverages various sensors, including cameras, LiDAR, and IMU, each contributing uniquely to the perception and understanding of the spatial environment. Cameras, both Red Green Blue (RGB) and depth‐sensing, provide visual and depth information critical for object recognition and scene structuring. LiDAR sensors offer precise distance measurements to surrounding objects, enhancing the device's ability to construct detailed 3D maps of its environment. IMUs, consisting of accelerometers and gyroscopes, track the device's motion and orientation, offering vital data to correct any drift in the device's estimated position over time.

The display technology in SC devices significantly impacts user experience by dictating the visual quality and immersion level of the virtual content. Micro organic light‐emitting diode (OLED) and micro light‐emitting diode (LED) display screens are at the forefront of this aspect, providing high resolution and pixels per inch (PPI), crucial for rendering sharp and compelling images (Hong et al., 2021; Huang et al., 2020). These display types are preferred for their ability to produce deep blacks and high contrast ratios, enhancing the depth and realism of the visual content. Furthermore, their low‐power consumption is vital for portable SC devices, extending battery life and enabling longer usage periods without the need for frequent recharging.

Another key hardware element is the optical system used to project virtual images into the user's field of view. The pancake lens, characterized by its thin and lightweight design, represents a significant advancement over the traditional Fresnel lens (Usukura et al., 2023). By utilizing a series of reflective surfaces, pancake lenses can achieve a compact form factor without compromising the field of view or image quality. This makes SC devices more comfortable to wear for extended periods, thereby increasing their practicality for everyday use.

The computing power required to process the complex algorithms of SC is provided by specialized hardware, including central processing units (CPU) and spatially designed chips. These components must handle vast amounts of data from sensors and execute SLAM algorithms in real time, necessitating high processing capabilities, low latency, and efficient power consumption. Spatially designed chips, often employing architectures optimized for parallel processing, are particularly effective in accelerating the computations needed for real‐time spatial mapping and localization.

In summary, the hardware components of SC devices form a synergistic system that enables sophisticated interactions with the physical world. From the precise environmental mapping afforded by SLAM technologies to the immersive visual experiences provided by advanced display technologies, and the computational backbone powered by specialized processors, each element plays a critical role in the functionality and applicability of SC. As these technologies continue to evolve, we can anticipate further enhancements in efficiency, performance, and user experience, expanding the possibilities of SC across various domains including but not limited to self‐driving, DT factory, and smart city.

## 3 APPLICATION OF SC IN PRECISION AGRICULTURE PRACTICES

SC is in the center of an agricultural revolution right now, which gets boosted by technologies of rapid progression and cutting‐edge nature, for instance, GIS, remote sensing (RS), GPS, the internet of things (IoT), DT, among others (Karada et al., 2023; Katkani et al., 2022; Omia et al., 2023). From simple GIS mapping and satellite imagery to the full scope of precision agriculture, this evolution is changing the face of farm management—soil and water quality analysis to crop health monitoring (Yousefi & Razdari, 2015). Thus, GIS forms the basis for SC in agriculture, giving out maps detailing the fine‐scale patterns of agricultural landscapes. It contributes to the provision of deeper insights into land usage, the type of soils present, and distribution of crops, which are essential for planning strategically and even administration of resources. For example, GIS improves irrigation and land management efficiency by location analysis to determine the best areas for growing various crops (Hu, 2023). In addition, RS compliments GIS through the supply of broader view of agricultural fields from above using satellite imagery and aerial photos in the monitoring of crop health and environmental conditions (Khanal et al., 2020). The preciseness of RS is of great help to precision agriculture, enabling a farmer to keep track and take care of the needs of crops across vast acreages over time (Shurlaeva et al., 2021; Zuckerman et al., 2024). These technologies have applications to guide operations of farm machinery for precision agriculture (Kumar et al., 2022). Leveraging SC technology, variable rate application (VRA) techniques can support strategies to predict more optimal application rates and times for seeds, fertilizers, and pesticides in specified areas in a field, thereby conserving resources and enhancing crop performance (Wang et al., 2023). The fusion of SC technology and IoT achieves another level to farm management: all components of a modern farm (e.g., lights, watering systems, feeders for farm animals, etc.) may be connected to sensors and networked devices. These devices allow for real‐time tracking of parameters such as soil moisture and crop growth stage. These types of data become critical for making informed decisions that will promote efficiency and environmental sustainability (Ndjuluwa et al., 2023; Thirisha et al., 2023).

The integration of GIS, RS, GPS, and IoT with SC technologies has begun to catalyze a paradigm shift in agricultural practices, enabling real‐time and comprehensive management insights. Such a holistic approach can significantly augment the precision and efficiency of farming operations, steering the agricultural sector toward sustainability and enhanced productivity to adapt to the dynamic global landscape (Hu, 2023; Verdouw et al., 2021). Utilizing real‐time data from GIS, RS, GPS, and IoT, DTs can construct 3D and intricately detailed virtual models of agricultural ecosystems. These models facilitate the visualization and scenario analysis crucial for optimizing strategies, managing resources, and forecasting yields (Pylianidis et al., 2021; Verdouw et al., 2021). Furthermore, as DTs continue to evolve into accessible virtual platforms for decision making, they will introduce revolutionary landscapes into precision agriculture and offer a comprehensive toolkit for predictive analysis and strategic planning for farming operations (Peladarinos et al., 2023; Purcell et al., 2023). For example, DTs have been reported to model the impact of varied agricultural practices, such as differing irrigation levels or pest management methods, and provide actionable insights for crop management (Nasirahmadi & Hensel, 2022). A case study from Midwest Brazil illustrates the transformative potential of these technologies when combined; GIS and RS were utilized for topographical and soil mapping, while GPS and IoT sensors monitored soil moisture and crop health, leading to significant yield improvements through optimized resource use (Poppiel et al., 2019). Similarly, a vineyard DT, integrating unmanned aerial vehicles (UAVs), 5G, edge/cloud computing, machine learning (ML), and AI, demonstrated sustainable agricultural practices including irrigation, pesticides, and fertilization (Edemetti et al., 2022). These case studies underscore the integrated application of DT and other technologies in agriculture, providing a holistic view of farming operations and enabling the modeling of various management strategies. In the future, SC in agriculture will enable even more interconnected, intelligent farming ecosystems to change farming practices. Continued advancements in AI and ML will enhance predictive behaviors for DT to produce accurate and precise models that can predict agricultural outcomes (e.g., yields and land use efficiency) under complex environmental conditions (Purcell et al., 2023). This will help in developing adaptable farm plans in the wake of climate change, fluctuating market scenarios, or varying availability of resources.

In addition to contributing toward the agricultural sustainability goals, increased efficiency and productivity with SC technologies holds potential to address the emerging global food security challenges (Wang et al., 2023). SC‐enabled optimization of resource use and waste reduction, coupled with increasing yields, is becoming more economically viable. The democratization of these technologies will help empower smallholder farmers in developing regions through data‐informed insights and precision management tools. For instance, affordable drone technology and open‐source GIS platforms enable farmers to access high‐resolution aerial imagery and soil health data, previously the domain of well‐resourced large agribusinesses. This access allows for tailored crop management strategies, optimizing inputs like water and fertilizers to increase yield without the need for large capital investments. An exemplary case is the project undertaken in sub‐Saharan Africa, where SC technologies facilitated the creation of detailed soil health maps. These maps were then used to implement precise farming techniques, significantly improving yields and sustainability for smallholder farms. To the extent that these benefits derive to any size of operation, they could make a difference in narrowing the productivity gap seen between the small and large farms, hence contribute to growth and equity in the sector, and enhance availability of food in the regions. Overall, SC in agriculture has an optimistic future, more likely to build a food system that is sustainable, productive, and resilient. Such a system will be well able to adapt to climate change and an increasing world population, ensuring the food security and sustainability of the food supplies of future generations.

## 4 APPLICATION OF SC IN SMART FOOD SUPPLY CHAIN

The food supply chain encompasses storage and distribution to consumers (Ahumada & Villalobos, 2009). SC applies to the food supply chain by enabling technologies that manage and optimize these processes in relation to their physical locations. It includes using GPS for tracking shipments, GIS for mapping supply networks, and AR for improving logistics and warehouse management. This technology helps in reducing waste, improving efficiency, and ensuring the timely delivery of fresh products to consumers by providing real‐time data and analytics on the spatial aspects of the supply chain. In this section we discuss the SC in warehouse management especially in layout planning, the use of AR and VR to enhance the order picking and delivery process, improve efficiency and reduce errors in food distribution networks, and ensure food freshness; and the distribution aspects including tracking and tracing products in the supply chain, real‐time monitoring and risk assessment, optimizing delivery routes, minimizing travel time, and specific applications for fuel consumption.

### 4.1 Warehouse management

**FIGURE 4:**
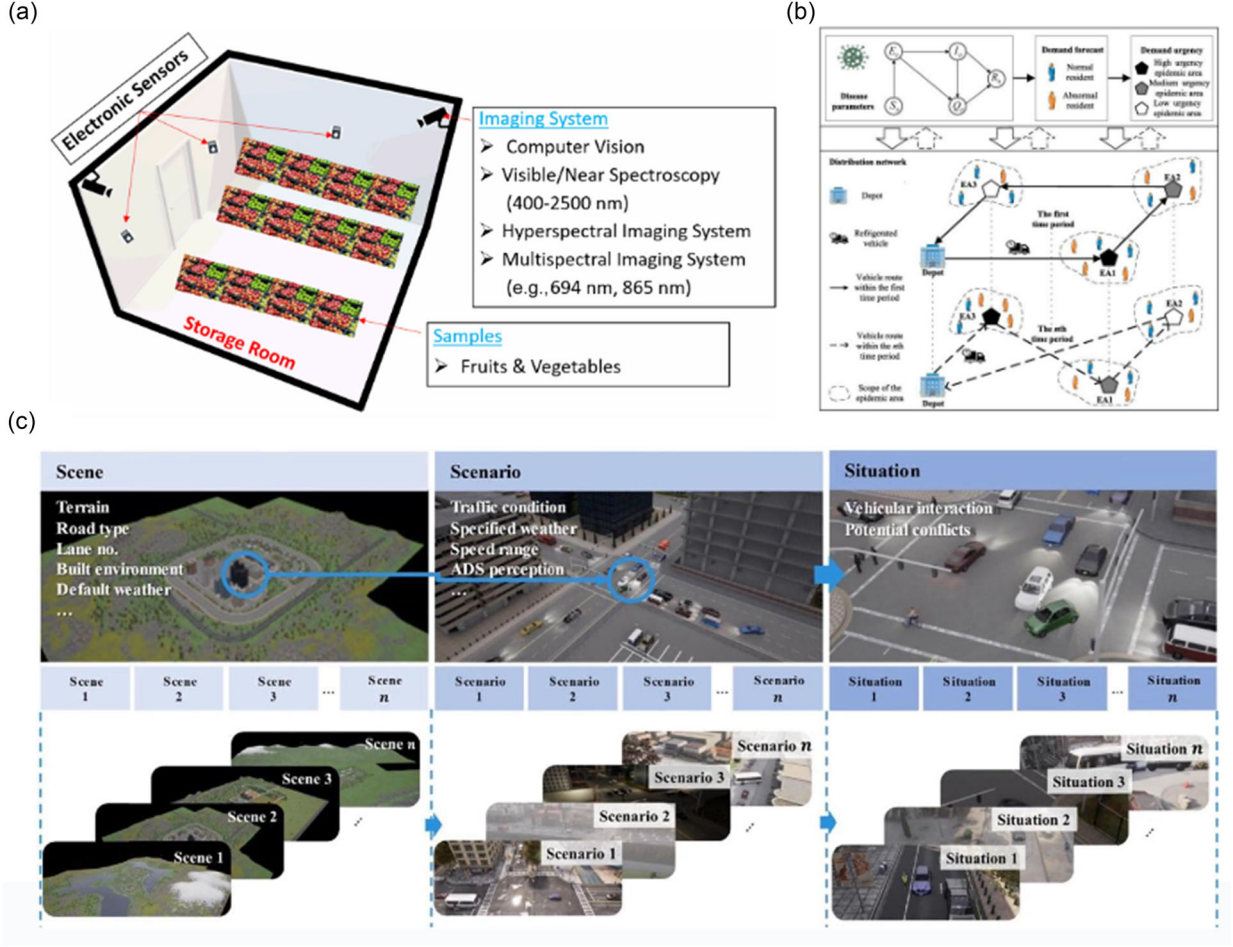
The application of spatial computing (SC) in smart food supply chain. (a) Schematics of multisensors coupled with imaging system for monitoring the quality of fruits and vegetables during cold storage (Onwude et al., 2020). (b) Illustration of a coordinated distribution scheduling delivery system (Zhang et al., 2024). (c) Essential virtual reality (VR) elements involved in the autonomous driving analytical framework (Xu et al., 2024).

Warehouse management is an important part of food supply chain management (Chen et al., 2020). Given the escalating complexity and efficiency demands in warehouse management, the food industry confronts challenges including effective space utilization, inventory accuracy, and labor shortages. Consequently, the integration of SC emerges as a critical solution, offering enhancements in layout refinement, process streamlining, and decision‐making capabilities, thereby significantly enhancing productivity and minimizing errors. Through the analysis of spatial data, warehouses can be designed to minimize travel time for pickers, alleviate bottlenecks for high‐demand items, and optimize layouts based on fluctuations in inventory levels or demand patterns (representative research was summarized in Figure 4). These strategies lead to substantial improvements in operational efficiency, encompassing accelerated order fulfillment, reduced labor costs, and enhanced overall productivity. For instance, a recent study investigated the application of a particle swarm optimization (PSO) algorithm to address the dynamic order batching and picker routing within a warehouse (Lin et al., 2024). This algorithm, by evaluating the “global worst” experiences and focusing on batch centers and order centers in 3D space, underscores the capacity of SC to substantially enhance order batching efficiency and picker routing. The optimization realized herein directly contributes to more streamlined warehouse operations, exemplifying SC central role in redefining logistics optimization. It is worth highlighting that transitioning toward SC technology with IoT architecture introduces unmanned vehicles, robots, and conveyor belts for internal warehouse transportation, promoting efficiency. Nagendra et al. (2019) proposed an IoT‐based framework for inventory control, involving process and domain model specifications alongside an information model detailing product positions and availability. This architecture, offering both automatic and manual operation modes, represents a Level‐4 IoT system with integrated devices and components for enhanced inventory management. Another recent study enhancing mobile robot navigation in warehouses through the utilization of model predictive control (MPC) and independent safety LiDAR systems (Sugimoto et al., 2024). Their study explored the integration of diverse travel strategies and safety measures to optimize robot trajectory planning and tracking. The innovation lies in the unique approach to bolster navigation safety and efficiency by harnessing SC and advanced sensing technologies, demonstrating considerable potential for creating efficient and secure warehouse layouts. In addition, an interesting study utilized similar technology within a grocery store management, which focused on optimizing shelf‐space allocation to guide in‐store traffic in a manner that maximizes expected impulse purchasing (Flamand et al., 2023). While specifically targeting retail environments, the principles delineated are applicable to warehouse management. SC is employed to allocate space efficiently, thereby influencing the movement and productivity of picking operations. The methodology's approach to optimizing layout for impulse purchasing indirectly emphasizes how SC can be leveraged to manage warehouse space more efficaciously, ensuring optimal placement of items for ease of access and streamlined picking processes. This optimization not only enhances efficiency but may also lead to increased satisfaction and reduced time in locating and moving items within a warehouse setting. In summary, SC is revolutionizing food warehousing by advancing spatial layout planning and general warehouse management. Through the strategic application of SC in optimizing warehouse layouts, warehouses are poised to achieve more efficient storage and retrieval systems, directly influencing productivity and operational costs.

To enhance the precision and efficiency of order picking processes within warehouses, AR technology serves as a pivotal innovation, providing warehouse workers with real‐time informational overlays at smart logistics network companies (Wang et al., 2020). The process involves capturing the user's viewpoint through a mini camera attached to head‐mounted displays (HMDs), with the captured image subsequently processed by a wearable personal computer (PC)—the nucleus of the AR system. This system identifies AR markers within the image, pinpointing their locations to superimpose additional data (such as text labels) accurately onto the real‐world image. The resulting composite image is then displayed through the HMD, offering enhanced navigational and informational support to the user. For example, a study innovated a scalable, wearable AR system designed to augment manual order picking operations through precise, floor‐related navigation (Fang & An, 2020). This system employs multimarker‐based global mapping to furnish continuous, accurate guidance, substantially reducing the cognitive load on warehouse personnel and improving both the speed and accuracy of order picking tasks. A recent similar report explored the application of Hololens2‐based AR technology to bolster efficiency in manual warehouse picking processes. Their study underscores AR's potential to minimize errors and expedite order fulfillment, verified through empirical testing and practical deployment. Additionally, Wang et al. (2020) emphasized mobile AR technology's burgeoning role in modern logistics (Wang et al., 2020). By leveraging mobile devices for object recognition, this technology overlays virtual information onto live video feeds, offering a novel approach to examining in‐house logistics operations. This research delineates the operational benefits of AR in logistics, including enhanced efficiency in inventory management, sorting, transportation, and resource allocation, presenting AR as a transformative tool for internal logistics and operation management. Collectively, these advancements signify a forward leap in integrating digital solutions into conventional warehouse settings, advocating for AR's capability to streamline operations, enhance accuracy, and mitigate errors through dynamic, visual guidance.

In addition to AR, SC technology drive automated guided vehicle (AGV) is another technology that collects the goods and prepares them for shipment. AGV is an autonomous robot designed to navigate through warehouses using predefined paths or advanced guidance systems with spatial data including radio waves, vision cameras, magnets, and lasers, play a critical role in streamlining the transport of goods. These vehicles reduce manual labor, lower operational costs, and boost productivity by performing tasks such as picking, packing, palletizing, and material transfer with precision and reliability. For example, SLAM‐based AGVs are capable of self‐navigation and map generation in environments devoid of prior mapping information, using mounted sensors for real‐time positioning and optimal path planning (Sun et al., 2022). Furthermore, the operational efficiency of AGVs can be augmented through visual SLAM, which employs cameras to construct detailed environmental maps. This process involves sensor data acquisition, visual odometry for estimating camera movement and local mapping, and nonlinear optimization to refine motion tracking and loop detection for revisiting previously mapped areas. A recent survey concluded that the application of visual SLAM facilitates precise local positioning of AGVs, enhancing their navigation capabilities within the warehouse. The introduction of AGV created new challenges such as path conflicts and system coordination in multi‐AGV environments necessitating innovative solutions with SC technology. One such approach involves an autonomous decentralized method for dynamic routing, wherein AGVs, acting as independent agents, identify optimal paths while incorporating collision avoidance mechanisms through negotiated route adjustments based on spatial data. This strategy ensures efficient and harmonious AGV operation, minimizing potential disruptions in the traffic system. Furthermore, the concept of AGV multi‐objective dynamic scheduling (AMODS) introduces a comprehensive framework for addressing the variability in AGV numbers and speeds. By leveraging a DT of the warehouse environment, AMODS facilitates a real time, bidirectional data exchange, enabling dynamic task allocation and optimized logistics system efficiency. This DT architecture encompasses the physical and virtual realms, ensuring a seamless integration of real‐world operations with virtual planning and control. Warehouse management vehicles take off from the ground into the air, fueled by SC technology. Apart from the previously mentioned UAV applications in precision agriculture, UAVs are also emerging for warehouse management tasks such as counting and localization. A study presented a novel low‐power, cost‐effective inventory management system using nano drones for small‐scale warehouses (Macoir et al., 2019). It integrated a hybrid path planning algorithm with dynamic obstacle avoidance using a Vector Field Histogram and a 2D‐LiDAR sensor, alongside a lightweight QR code detection for product inventory. The system achieved a 92% accuracy in QR code detection and 81% accuracy in product localization with a dead reckoning algorithm, demonstrating its feasibility and efficiency in cluttered warehouse environments. Together, these technological advancements underscore the transformative potential of AGVs and UAVs in optimizing warehouse operations, from improving the accuracy and speed of order fulfillment to innovating inventory management practices.

Taking the advantage of SC technology, VR has applications for risk‐free training of employees, facilitating the acquisition and honing of skills pertinent to warehouse operations—such as picking, packing, and adherence to safety protocols—without necessitating physical resources (Zawadzki et al., 2020). VR is increasingly instrumental in layout planning and optimization, as it allows for the simulation of warehouse operations. This enables managerial personnel to evaluate and refine workflows sans interruption to actual warehouse functions. A study developed a VR training platform that seamlessly integrates with an extant warehouse management system, aiming to impart training on standard order picking processes (Yigitbas et al., 2020). This platform simulates an operational warehouse environment replete with actual stock and orders, with preliminary usability assessment results indicating promising training outcomes and high end‐user acceptance. WareVR, a DT of a warehouse environment, not only visualizes robotic systems but also incorporates a real‐time video feed from a UAV‐mounted camera (Kalinov et al., 2021). The WareVR interface affords operators the capacity for comprehensive stocktaking, remote inventory management, and UAV teleoperation for meticulous inspection, thereby enhancing the operator's engagement with the inventory process through intuitive and efficient UAV interaction (Grandi et al., 2023). Collectively, these studies underscore VR's pivotal roles in advancing warehouse management practices, offering innovative solutions for training, operation optimization, and remote inventory management.

### 4.2 Route optimization

In the current context of the food supply chain, the paramount importance of ensuring traceability and freshness for food products, as opposed to other types of goods, has mandated the adoption of sophisticated technological solutions. Among these, the integration of IoT devices, such as GPS and cameras, along with the conceptualization and implementation of DT, stands at the forefront of innovations enhancing the precision in tracking food products from origin to consumer (Wu et al., 2022). Food supply chain already utilized GPS technology for real‐time meticulous tracing of products across various supply chain stages. A recent study proposed the dynamically partitioned self‐adaptive tracing and tracking (DPSTT) system, leveraging Bayesian estimation for dynamic sampling and partitioning to trace and track food products with high efficiency (Nagarajan et al., 2022). This innovative system, characterized by its use of directed acyclic graph (DAG) and depth‐first search (DFS) traversal schemes, demonstrated a notable tracing accuracy of 95.3% at a minimal sampling rate of 7.6%, surpassing traditional global sampling methodologies.

Furthermore, the role of DT technology transcends mere tracing and tracking, extending into the domain of postharvest engineering where the freshness and quality of food products are of paramount concern (Onwude et al., 2020). DT technology, enriched with sensor‐derived real‐time monitoring capabilities, offers a robust framework for diagnosing and prognosticating potential issues along the supply chain that may escalate food losses. These challenges, ranging from physiological and hygrothermal to biotic and mechanical effects, necessitate a multidisciplinary approach to quality and shelf‐life management. The development of DT for mango showcased the application of holistic models, including heat and mass transfer (HMT) and kinetic models of ripening (KMR), to assess and predict the evolution of quality attributes such as firmness, soluble solids content, and vitamin content (Tagliavini et al., 2019). Utilizing input temperature data from the mango cold chain, these models facilitate the creation of a virtual mango fruit DT, enabling predictive quantification of fruit quality across multiple shipments. In essence, the integration of IoT and DT within the food supply chain represents a significant stride toward achieving enhanced traceability, ensuring product freshness, and ultimately fostering a more sustainable and efficient supply chain ecosystem.

Innovative strategies are needed to overcome challenges related to last‐mile food delivery, labor shortages, and logistical issues in servicing remote areas. Integration of SC technologies—encompassing AGV, UAV, AI, IoT, blockchain, and DT—emerges as a transformative strategy to augment the efficiency, sustainability, and safety of food distribution networks. A recent study introduced an IoT‐based food supply with dynamic vehicle routing (IFSCDVR) using Bee Colony algorithm, facilitating real‐time monitoring and quality assurance of food items through an extensive sensor network (Nagarajan et al., 2022). This network feeds data to a control and monitoring system (CMS), which aggregates and analyzes the information, further enhancing traceability and safety protocols via SC. To optimize transportation routes and mitigate traffic congestion, the study proposes a novel vehicle routing mechanism that utilizes GPS data to generate efficient pathways, thereby integrating Google traffic data for real‐time route adjustments. In addition, to mitigate ground transportation's limitations, UAVs have been spotlighted as a viable alternative for food distribution, particularly in reaching remote or geographically challenging areas. A study showcased a UAV simulation for optimizing food delivery, demonstrating the capability of drones to manage high‐volume deliveries efficiently (Kelemen & Szénási, 2023). With the simulation, it was possible to perform a run where orders were continuously generated and drones allocated to destinations, thus realizing the collaboration of up to 100 drones and the simultaneous delivery of over 1500 orders. UAVs, characterized by their ability to bypass conventional traffic and geographical constraints, offer a promising avenue for rapid, low carbon footprint delivery solutions and personalized delivery experiences. Despite anticipated challenges related to regulatory, climatic, and operational capacity considerations, drones are a promising technology that will revolutionize food distribution.

## 5 APPLICATION OF SC IN FOOD R&D AND MANUFACTURING

Major challenges in food manufacturing and R&D include the necessity to amplify operational efficiency, guarantee food safety, adhere to regulatory requirements, and cater to the burgeoning demand for personalized and health‐centric products (Lillford & Hermansson, 2021). SC technologies emerge as quintessential tools to meet the moment, offering the capability to facilitate immersive training experiences for personnel, simulate intricate production and refinement of R&D processes, and enable remote maintenance and quality assurance inspections. By integrating these technologies, food manufacturing and R&D sectors are poised to transcend traditional limitations, fostering enhanced process efficiency, innovation, and compliance with safety standards (Galanakis, 2020).

SCs are transforming the food sector employee domain by delivering immersive and risk‐free environments for efficient personnel training. Evidently, the integration of VR and AR into training processes offers numerous advantages, including enhanced cognitive performance, increased safety, and improved learning retention. For example, VR immerses users in a fully virtual environment, making it particularly useful for simulating manufacturing tasks, safety training, and complex machinery operation without the associated risks. It allows for the replication of real‐world scenarios where learners can practice and hone their skills. A study assessed the effectiveness of VR versus traditional video training for fire extinguisher use in food manufactory, focusing on the pull, aim, squeeze, sweep (PASS) procedure. Utilizing a pretest, post‐test, and retention test framework with 93 participants, the research found VR training led to significantly better immediate knowledge acquisition and long‐term retention compared to video training. Specifically, VR trainees demonstrated a higher level of knowledge retention 3–4 weeks post‐training, with an 81% localization accuracy using a dead reckoning‐based algorithm for product identification. Additionally, VR training resulted in a more substantial increase in self‐efficacy immediately after training, which was maintained over time, unlike the video group that saw a significant drop. The study underscores VR's potential as a superior method for emergency preparedness training, offering a more engaging, effective, and retained learning experience. Moreover, AR enhances the user's real‐world perception by superimposing digital information. In manufacturing training, AR offers significant advantages by guiding workers through assembly, maintenance, and providing instant data overlays. This direct integration with the physical workspace reduces errors and increases efficiency, leveraging the real environment unlike VR's entirely simulated settings. The study examines the integration of AR in the food industry as a part of Industry 4.0, highlighting its potential to improve safety, efficiency, and innovation amid challenges like privacy, social acceptance, and technological limitations (Jagtap et al., 2021). It proposes a framework for AR implementation, focusing on enhancing transparency and efficiency while addressing the sector's challenges related to climate change, consumer demands, and regulations. Overall, the implementation of SC technology in manufacturing training offers notable advantages over conventional methods, including an enhanced learning experience through immersive and interactive environments, which fosters improved engagement and comprehension of intricate processes. SC technology ensures safety by enabling training within a risk‐free virtual space, particularly for hazardous tasks. It also reduces costs by minimizing the need for physical prototypes and materials, and offers unparalleled flexibility and accessibility, allowing for efficient training of a geographically diverse workforce. It is worth to highlight that the integration of SC technology with AI holds a promising future for crafting personalized learning experiences, leveraging advanced algorithms and immersive environments to tailor educational content and interactions to individual learner needs and preferences.

Another emerging application of SC in food manufacturing is DT. By harnessing the power of DT, food manufacturers can simulate, predict, and control the physical counterparts with remarkable accuracy, thus enabling optimized performance and innovation. For instance, employing integrated processes and DT models has been reported to enhance the manufacturing processes in the food industry, focusing on process simulation and production scheduling. DT simulations can identify key challenges and opportunities in food processing industries (FPIs), such as product safety, cost minimization, and the need for timely delivery amid uncertainties. By applying these models to a large‐scale brewery case study, the research demonstrates potential benefits like improved efficiency and reduced costs. The study concludes with the proposition that adopting digital modeling approaches significantly benefits FPIs by optimizing design and operation levels, thereby contributing to more efficient, flexible, and profitable manufacturing processes.

## 6 APPLICATION OF SC AT THE STAGE OF CONSUMERS

SC fundamentally redefines consumer feedback mechanisms through the integration of immersive technologies, such as AR and VR, enhancing the granularity and scope of consumer insights beyond traditional methods. This evolution facilitates the acquisition of comprehensive data, encompassing not only conventional verbal and written feedback but also capturing nuanced nonverbal cues, including gestures and movements. Such multidimensional data collection provides a profound understanding of consumer behavior, enriching the feedback with contextually rich, simulated real‐world environments. The latest research highlights the multifaceted applications of VR, from product design and development to advertising and the hospitality industry. Utilizing a robust methodological framework, Kim and Choo (2023) measured perceptual curiosity (*α* = 0.933) and consumer creativity via divergent thinking and creative dressing tasks, exploring the nuanced interplay between VR experience mode (nonimmersive vs. immersive) and store fantasy (reality‐based vs. fantasy‐based; Kim & Choo, 2023). The findings elucidate a significant relationship where immersive VR experiences heighten perceptual curiosity more effectively than nonimmersive ones. Moreover, the presence of store fantasy significantly amplifies this effect, fostering a conducive environment for consumer creativity. This research demonstrates how VR shopping experiences enhance consumer creativity by mediating the role of perceptual curiosity, especially emphasizing the impact of immersive versus nonimmersive environments and the influence of store fantasy. Another study investigated how consumers assess product packaging in a virtual environment compared to physical handling. It explores whether VR could effectively simulate the sensory and structural cues of packaging, influencing consumer perceptions of sustainability and willingness to pay a premium (Chiu et al., 2021). Through focus groups, experiments, and conjoint analysis, the findings suggest that certain packaging materials, like glass, and tactile features, such as rough textures, significantly enhance perceived sustainability and premium payment willingness, with VR evaluations closely aligning with real‐life responses. This research marks a significant step forward in understanding how digital environments can mimic real‐world shopping experiences, providing a reliable method for testing packaging options in a controlled, immersive setting. These studies collectively underscore VR's transformative role in simulating real‐world shopping experiences and fostering consumer engagement and creativity, marking significant advancements in digital consumer interaction research. The implications of these findings are vast, suggesting that as VR technology continues to evolve, its application across industries will likely expand, offering even deeper insights into consumer behaviors and preferences.

AR is also rapidly becoming a cornerstone technology in the realm of consumer feedback and retail marketing, offering unprecedented opportunities to enhance consumer engagement and transform retail experiences. The latest research across various sectors—from fashion retail to e‐shopping—provides compelling insights into how AR technology can reshape consumer interactions, satisfaction, and expectations. A study introduces a novel system leveraging spatial augmented reality (SAR) and deep learning for emotion recognition to enhance customer satisfaction in restaurants (Soon et al., 2023). This system dynamically assesses user experiences via facial expressions, optimizing the dining environment based on real‐time feedback. Experimental evaluations under varied lighting and viewing angles identified optimal conditions for SAR applications, showing a significant improvement in customer satisfaction. Survey results indicated high satisfaction with the 3D virtual experience, underscoring the system's potential to transform restaurant customer interactions by providing personalized, interactive dining experiences and integrating customer feedback into logistical and decision‐support systems for improved service delivery. The study employed a structured online survey targeting AR application users in Shanghai's coffee shops to examine the DeLone and McLean Information System Success Model ISSM within the context of AR retail applications in the retail food chain. By integrating system, service, and information quality as predictors, the research explored their influence on user satisfaction and continuance intention, finding that all three quality dimensions significantly impact user satisfaction and intention to use AR retail applications (ARRA), thus underscoring the importance of these factors in enhancing user benefits and satisfaction with ARRA in the retail food industry. Together, these studies comprehensively demonstrate AR's transformative potential in retail and consumer experiences. By enhancing marketing strategies, deepening consumer engagement through immersive experiences, and reshaping consumer expectations and satisfaction levels, AR stands out as a pivotal technology in the current and future landscape of retail.

The integration of 3D reconstruction and AI into the field of nutritional science represents a significant leap forward in dietary assessment and food quality evaluation. Recent research underscores the transformative potential of these technologies, offering more accurate, efficient, and user‐friendly methods for determining nutritional values, estimating food volumes, and assessing diet quality (Table 1). Ma et al. (2022) present a deep learning approach for estimating the nutritional content of Chinese market foods from images. Utilizing the ChinaMartFood‐109 dataset, which comprises images of 109 food types with associated nutrient information, the research employs CNNs to predict nutrient values (Ma et al., 2022). Results demonstrated that different nutrients (calories, protein, fiber, thiamine, and iron) exhibited variable estimation accuracy across the methods. Subsequently, the goFOOD™ system (https://go‐food.tech/) is designed to translate food images to nutrient information, which marks a notable advancement in dietary assessment (Lu et al., 2020). By employing AI and 3D reconstruction algorithms, this system aims to significantly enhance the accuracy of nutritional information estimation from images, promising improved outcomes for diet tracking and health management. A study tested this system on Mediterranean diet (Konstantakopoulos et al., 2023). The result showed that it can recognize single meal photo of food and drink items, estimate the serving size, automatically calculate a Mediterranean diet adherence score, and generate a weekly feedback report. Meanwhile, this system, integrated into a smartphone app, was also tested for its feasibility and accuracy against dietitian assessments and received positive feedback from users for its ease of use and helpfulness in diet monitoring. This approach illustrates the potential of leveraging technology to refine dietary assessment tools, making them more reliable and effective for users. Lo et al. (2020) reviewed related work and stated that image‐based techniques, including 3D reconstruction, can be effectively applied for food classification and volume estimation (Lo et al., 2020). These technics can overcome challenges like view occlusion and scale ambiguity, offering a significant improvement over existing methods. They highlight the significance of processing speed, model accuracy, efficiency, and the constraints of current methods, alongside the promise of deep learning techniques in enhancing dietary assessment accuracy. Moreover, another survey comprehensive overview of vision‐based dietary assessment (VBDA) technologies, focusing on advancements in computer vision and machine learning for automatic food recognition, volume estimation, and nutrient analysis (Wang et al., 2022). It highlights the shift from traditional dietary assessment methods to more accurate and less time‐consuming AI‐driven approaches. The paper discusses both multistage and end‐to‐end VBDA architectures, evaluates existing datasets, and outlines future challenges and directions in the field, aiming to encourage further research and development of practical VBDA solutions, emphasizing the significance of 3D reconstruction for food volume estimation, underlining the crucial role of technological advancements in achieving precise nutritional evaluations. Recently, a representative work, FVEstimator, was developed for estimating the volume and caloric content of food (Kadam et al., 2022). The model was trained on eight different classes of Indian breakfast foods of all shapes, demonstrating a precision of more than 98.5% for regular shaped food items (Zhang et al., 2023). This advancement offers significant potential for dietary monitoring and health management, addressing challenges in accurately measuring food volume and calorie intake.

**TABLE 1:** Summary of food volume and nutrients estimation.

Study	Data	Food type	Model categories	Results	Refs.
Improved voxel‐based volume estimation and pruning severity mapping of apple trees	365 items, images	Apple trees	Voxel‐based volume calculation algorithm	Coefficient of determination 0.994; Mean absolute percentage error 2.919%	Dong et al. (2024)
Attention‐driven active vision for efficient reconstruction of plants	27 items, image	Tomato plants	Active vision strategies, next‐best‐view (NBV)	Accuracy up to 0.91	Burusa et al. (2022)
foodCAM: Mobile application for estimating caloric intake using deep learning	Five items, image	Various, mainly cafeteria foods	Deep learning, convolutional neural network (CNN)	Average accuracy of 94.4%	Amugongo et al. (2023)
A vision‐based method to estimate volume and mass of fruit/vegetable: Case study of sweet potato	20 items, light detection and ranging (LiDAR) + image	Sweet potato	Deep learning, CNN	Accuracy of up to 96% with an *R* ^2^ of 0.98	Huynh et al. (2022)
Food volume estimation by multilayer superpixel	Seven items, image	Various	Multilayer segmentation, SLIC	Error rate 0.58%–34.2%	Zheng et al. (2023)
Food volume estimation by integrating three‐dimensional (3D) image projection and manual wire mesh transformation	26 items, image	Various	3D reconstruction	Error rate 34.2%–56.1%	Smith et al. (2022)
A novel approach to dining bowl reconstruction for image‐based food volume estimation	228 items, image	Various, in dining bowls	Dining bowl reconstruction	Error rate 0.2%–10.6%	Jia et al. (2022)
Research on 3D reconstruction method and application of food in stroke patients based on RGB‐D image	RGB‐depth imaging	Food for stroke patients	3D reconstruction	Average error rate 3.89%	Yuan et al. (2023)
Measuring food volume from RGB‐depth image with point cloud conversion method using geometrical approach and robust ellipsoid fitting algorithm	126 items, RGB‐depth imaging	Various	Point cloud conversion, ellipsoid fitting	Absolute relative error 2.6%–4.4%	Sari and Gofuku (2023)
Volumetric food quantification using computer vision on a depth‐sensing smartphones	128 items, image	Various	Deep learning, CNN	Absolute error: 35.1 g	Herzig et al. (2020)
goFOOD(TM): An artificial intelligence system for dietary assessment	319 categories, image	Dishes	Deep learning, CNN	Pearson correlations 0.4–0.92	Lu et al. (2020)
Classify and estimate the volume and nutritional content of Mediterranean food dishes using images	148 items, image	Dishes	Deep learning, CNN	Average error rate 10.5%	Konstantakopoulos et al. (2023)

Collectively, these studies paint a promising picture of the future of nutritional evaluation and food analysis. By harnessing the power of 3D reconstruction and AI, researchers and developers are paving the way for more precise, efficient, and accessible dietary assessment tools. These advancements hold the potential to revolutionize nutritional science, offering new avenues for clinical and personal health management that are grounded in accuracy and ease of use.

## 7 ONGOING CHALLENGES

### 7.1 High implementation costs

The implementation of SC applications within the food industry encapsulates a substantial financial commitment, attributed to multifaceted factors ranging from advanced hardware requisites to software development, content creation, system integration, and requisite training and support mechanisms. The deployment of SC technologies necessitates the acquisition of specialized hardware, such as headsets, sensors, and wearable devices, which are inherently expensive due to their need for high‐resolution displays and sophisticated computing capabilities to render immersive experiences. Additionally, development and customization of software for these SC applications require a significant investment of resources and time. This encompasses expenses related to hiring proficient developers, procuring software licenses, and the continuous troubleshooting for maintenance and updates essential for compatibility with evolving hardware and operating systems. The creation of engaging and realistic content further escalates costs, requiring the expertise of graphic designers, animators, and content creators to produce high‐quality 3D models, animations, and interactive elements. Integration of SC technologies with existing IT infrastructures and business processes in the food industry introduces additional complexity and costs, necessitating system modifications, data compatibility assurance, and employee training for effective technology utilization. Moreover, comprehensive training for employees is imperative to ensure proficient use of these technologies, thereby adding to the total cost of ownership and highlighting the substantial financial investment required for the implementation of SC applications in the food industry.

### 7.2 User acceptance and experience

User acceptance and experience within the realm of SC in the food industry is significantly influenced by an array of technical and ergonomic factors that hinder the optimal utilization and immersive engagement of these technologies. Key technical factors such as low‐resolution displays compromise the visual fidelity of virtual and AR environments, detracting from user engagement by impeding the creation of immersive and visually compelling experiences. High latency, characterized by the delay between user actions and system responses, disrupts immersion and induces disorientation, underscoring the importance of minimizing latency to maintain a seamless interactive experience. Furthermore, the accuracy of tracking user movements and behavior is paramount; inaccuracies can lead to issues that will severely diminish the user's sense of presence within the virtual or augmented environment. Additionally, the user experience is constrained by hardware limitations, including a restricted field of view in VR headsets or AR glasses, which limits peripheral vision and reduces spatial awareness, and poor battery life in wearable devices, which hampers extended use and mobility. Ergonomic design issues, such as the weight and comfort of SC devices, also play a critical role in user acceptance, where discomfort can lead to rapid user fatigue and decreased engagement. Software and content limitations, including the scarcity of high‐quality content and software optimization issues, further impede user satisfaction. Lastly, health and safety concerns, such as eye strain, motion sickness, and physical discomfort from prolonged use of VR and AR technologies, present significant challenges that the industry must address. These factors collectively highlight the complexities involved in enhancing user acceptance and experience in SC applications within the food industry, necessitating a holistic approach to technological development and user‐centric design.

### 7.3 Supply chain integration

SC technologies integration introduces complex data privacy and ethical challenges that necessitate rigorous scrutiny and thoughtful management. One of the paramount concerns is the adherence to strict data protection regulations, given the extensive collection and processing of both personal and operational data inherent in SC applications. Ensuring compliance with comprehensive frameworks like the General Data Protection Regulation (GDPR) in Europe is vital, as these regulations mandate stringent data security and privacy protections. Moreover, the ethical implications extend beyond mere regulatory compliance, touching upon the transparency of data usage, potential surveillance concerns, and the risk of infringing on individual rights and autonomy. It is imperative for entities deploying SC in the supply chain to establish transparent communication channels with all stakeholders, clearly articulating the purpose, scope, and methods of data collection and processing. This transparency is essential not only for ethical reasons but also for fostering trust and acceptance among users and stakeholders. Furthermore, ethical challenges also encompass the proactive consideration of the broader societal impacts of these technologies, such as the potential for job displacement due to automation and the environmental toll of increased energy consumption and electronic waste. Addressing these challenges requires a comprehensive and multidisciplinary approach, combining technological innovation with robust ethical guidelines and privacy safeguards to ensure that the integration of SC into the food supply chain is both responsible and sustainable.

### 7.4 Neophobia

The phenomenon of neophobia, characterized by a reluctance or fear of engaging with new technologies, presents a significant impediment to the widespread adoption and satisfaction of SC technologies within the business sector. This resistance is amplified by the intrinsic novelty of SC, which diverges markedly from traditional business methodologies and tools, thereby not only challenging the notion of product‐market fit but also introducing substantial marketing and adoption obstacles. According to insights from Deloitte's NExT team, this trepidation toward unfamiliar technology constitutes a formidable barrier, hindering the progression of SC from its current status as a niche innovation to reaching broader markets and public acceptance. Despite the growing awareness of SC's potential, the depth of its transformative impact on business operations and workflows remains largely underappreciated, with a significant number of enterprises lacking preparedness to navigate the disruptive shifts it promises. Having limited familiarity with these technologies often leads to perceptions of undue complexity, deterring potential adopters. Moreover, apprehensions regarding the usability of these novel technological interfaces further exacerbate user engagement challenges. Overcoming these barriers necessitates a multifaceted approach, emphasizing targeted educational initiatives to demystify SC, the development of user‐centric design principles to enhance interface accessibility, and the establishment of robust support frameworks. Such measures are crucial for facilitating a more seamless transition toward the integration of SC technologies into conventional business practices, thereby promoting wider acceptance and utilization.

## 8 PERSPECTIVES FOR SC APPLICATIONS TO MEET FOOD INDUSTRY NEEDS

### 8.1 More efficient agricultural production

The integration of SC into controlled environment agriculture (CEA), such as vertical agriculture, and urban farming represents a transformative approach toward optimizing land use and enhancing resource efficiency (Galanakis, 2024). By employing AR and VR technologies, growers can design and manage cultivation spaces with unprecedented precision, maximizing yields in confined spaces. These technologies facilitate the meticulous planning and execution of vertical farming structures, which stack cultivation layers vertically to conserve space and resources. This method not only bolsters production in limited areas but also significantly reduces the environmental footprint of agricultural practices, contributing to the development of sustainable urban food systems. AR and VR tools can assist in visualizing and simulating crop growth under various controlled conditions, allowing for the adjustment and fine‐tuning of factors such as lighting, water, and bioavailable nutrient levels to optimize plant growth and yields without the expansive land use that traditional agriculture requires. Furthermore, the application of SC through RS and analysis heralds a new era in precision agriculture, making farming practices more sustainable and efficient. Drones and satellites equipped with sophisticated sensors gather detailed spatial data on crop health, soil conditions, and moisture levels. This granular data enable farmers to implement precise agricultural interventions, targeting specific areas for treatment rather than employing broad‐spectrum approaches. Such targeted interventions can significantly reduce the unnecessary application of pesticides and optimize the use of water and fertilizers, thereby minimizing the environmental impact. Additionally, SC technologies offer robust educational platforms, streamlining the dissemination of best practices and innovations in crop management. Through virtual simulations and real‐time data analysis, farmers can receive tailored training and advice, enhancing productivity and sustainability in agricultural operations.

### 8.2 More resilient supply chains

SC technology is revolutionizing the realm of food traceability and transparency, offering innovative solutions for supply chain optimization and risk management (Galanakis, 2023). This advanced approach leverages spatial data analysis across the supply chain to predict and mitigate potential disruptions in real time. By identifying potential bottlenecks or disruptions before they occur, companies will be able to optimize routing and logistics for enhanced efficiency and sustainability. For example, SC may help facilitate the development of sophisticated monitoring systems capable of real‐time detection of biological and/or chemical signatures for pathogens, toxins, or contaminants in food products. Integrating sensor data with spatial analytics, these systems can trigger instant alerts, enabling prompt actions to uphold food safety and quality standards. Furthermore, the integration of SC with blockchain technology significantly enhances food traceability systems. This combination allows for the tracking of spatial data of food items throughout the supply chain, securely recording this information on a blockchain. Such integration has applications for reaching unparalleled transparency and traceability, aiding in quickly pinpointing contamination sources, efficiently managing recalls, and bolstering consumer trust by offering a transparent view of the product's journey from “farm to fork.” Another notable example of SC's potential is precisely locating the transmission pathways of different pathogens as they may spread throughout the supply chain, offering a detailed and actionable understanding of contamination sources and their propagation routes. By creating a virtual replica of the supply chain within smart city management, stakeholders can simulate scenarios, predict outcomes, and implement strategies to prevent or contain potential contamination, thereby ensuring the safety and integrity of food products. This sophisticated use of technology not only optimizes supply chain operations but also significantly advances food safety protocols, providing a robust framework for risk management and quality control in the food industry.

### 8.3 More sustainable food manufacturing

SC is poised to significantly impact sustainable food manufacturing, offering innovative solutions for waste reduction, the advancement of a circular economy, and the enhancement of research and educational methodologies. By leveraging the capabilities of SC to track and analyze the flow of food products throughout the manufacturing process, companies can pinpoint areas where waste is generated at disproportionate rates. This granular visibility enables manufacturers to refine their production processes, thereby minimizing waste and optimizing resource use. For example, SC can identify excessive packaging materials or inefficient food processing methods that contribute to waste, prompting the adoption of more sustainable practices. Moreover, this technology can orchestrate the efficient redirection of surplus food from producers to consumers or charitable organizations, addressing food insecurity while reducing waste. In the realm of food science research, SC introduces the concept of virtual laboratories, a groundbreaking development that facilitates global collaboration among scientists. These virtual labs simulate real‐world experimental environments, allowing researchers to conduct tests and share results in real time without the constraints of geographical boundaries or the need for extensive physical resources. This approach not only accelerates the pace of innovation in food technology but also significantly reduces the carbon footprint associated with traditional research practices. Scientists can virtually experiment with new food preservation techniques or sustainable packaging solutions, fostering a collaborative and eco‐friendly research culture. Furthermore, SC transforms educational platforms in food science and technology. Through immersive VR experiences, students can delve into the intricate world of biochemical processes, food engineering, and safety protocols in an engaging and interactive manner. This hands‐on learning approach enhances comprehension and retention of complex concepts, preparing students for careers in the food industry with a strong foundation in sustainability principles. Virtual tours of manufacturing facilities, interactive simulations of food processing techniques, and 3D visualizations of molecular structures are just a few examples of how SC can revolutionize education in this field, inspiring a new generation of innovators committed to sustainable food production.

### 8.4 More active consumer engagement

SC will undoubtedly revolutionize consumer engagement within the food sector by offering immersive and interactive experiences that illuminate the journey from farm to fork. Through AR applications, consumers can gain insights into the origin, path, and nutritional aspects of their food products. By scanning product labels, individuals are not just informed about the nutritional facts but are also transported on a virtual journey that highlights the product's supply chain, including the cultivation practices and the communities involved in its production. This immersive storytelling not only enhances consumer knowledge and connection with the food they consume but also promotes transparency and trust in food brands. Furthermore, AR applications can extend into “smart kitchens,” introducing interactive environments where recipes and cooking guidance are augmented onto the physical space. Users would be able to receive visual step‐by‐step instructions and essential information, such as portion sizes and nutritional content, directly in their cooking area, making meal preparation both engaging and educational. In addition to enhancing consumer engagement through interactive experiences, SC integrates with health and wellness platforms to offer personalized nutrition and lifestyle recommendations. By analyzing spatial data on users' physical activities, dietary intake, and health metrics, these platforms can provide tailored advice aimed at improving overall well‐being. This bespoke approach to nutrition is further enriched with gamification and educational outreach, utilizing engaging experiences to motivate individuals toward sustainable eating habits and informed food choices. The potential of SC extends to the creation of virtual laboratories in the food science sector, enabling scientists worldwide to collaborate in real time, conduct experiments, and share findings without geographical constraints. This not only accelerates innovation but also fosters a global community of researchers focused on advancing food science and technology. By leveraging SC, the food industry can enhance consumer engagement, promote sustainable practices, and foster an informed and health‐conscious consumer base, ultimately supporting overall well‐being.

## 9 CONCLUSION

SC technologies, including AR, VR, and DTs, offer significant potential to revolutionize the food supply chain by improving operational efficiency, sustainability, and safety. This review highlighted SC's ability to address major challenges, such as logistical inefficiencies, quality control issues, and lack of traceability, thus enabling advancements in precision agriculture, enhanced supply chain visibility, streamlined food manufacturing, and more personalized consumer interactions. The findings suggest that while SC technologies can profoundly transform the food supply chain, their widespread adoption faces several obstacles. These include technical limitations such as high implementation costs, integration complexity, and insufficient industry‐wide standards. Privacy concerns and the need for comprehensive regulatory frameworks are additional barriers that must be addressed. Implications of these findings indicate that SC can play a critical role in creating more resilient, transparent, and sustainable food systems. However, these benefits can only be fully realized through further interdisciplinary research and collaborative efforts across industries. Limitations of the review include a focus on general applications, without in‐depth case studies of specific SC deployments. Additionally, the rapidly evolving nature of SC technologies may mean that some recent advancements were not captured. Future research should explore more specialized SC applications in food systems, address privacy and security challenges, and develop standardized frameworks for regulatory compliance. Empirical case studies would also provide valuable insights into real‐world adoption. In conclusion, SC has the potential to reshape the global food supply chain, but its success will depend on overcoming current challenges and fostering innovation through collaborative research and policy development.

## AUTHOR CONTRIBUTIONS

**Peihua Ma**: Conceptualization; data curation; visualization; writing—original draft; formal analysis; investigation. **Xiaoxue Jia**: Data curation; writing—original draft; visualization; investigation. **Mairui Gao**: Writing—original draft; data curation; investigation. **Zicheng Yi**: Data curation; writing—original draft. **Shawn Tsai**: Data curation; writing—original draft. **Yiyang He**: Data curation; writing—original draft. **Dongyang Zhen**: Data curation; writing—original draft; validation. **Bei Fan**: Writing—review and editing. **Fengzhong Wang**: Writing—review and editing. **Ryan A. Blaustein**: Supervision; writing—review and editing; resources. **Qin Wang**: Supervision; writing—review and editing. **Cheng‐I. Wei**: Project administration; supervision; writing—review and editing; funding acquisition.

## CONFLICT OF INTEREST STATEMENT

The authors declare no conflicts of interest.

## References

[crf370055-bib-0001] Abadi, M. , Agarwal, A. , Barham, P. , Brevdo, E. , Chen, Z. , Citro, C. , Corrado, G. S. , Davis, A. , Dean, J. , Devin, M. , Ghemawat, S. , Goodfellow, I. J. , Harp, A. , Irving, G. , Isard, M. , Jia, Y. , Józefowicz, R. , Kaiser, L. , Kudlur, M. , … Devin, M. (2016). Tensorflow: Large‐scale machine learning on heterogeneous distributed systems. *arXiv preprint arXiv:1603.04467*.

[crf370055-bib-0002] Ahumada, O. , & Villalobos, J. R. (2009). Application of planning models in the agri‐food supply chain: A review. European Journal of Operational Research, 196(1), 1–20.

[crf370055-bib-0003] Amugongo, L. M. , Kriebitz, A. , Boch, A. , & Lütge, C. (2023). Mobile computer vision‐based applications for food recognition and volume and calorific estimation: A systematic review. Healthcare, 11(1), 59. 10.3390/healthcare11010059 PMC9818870 36611519

[crf370055-bib-0004] Bradski, G. (2000). The opencv library. Dr. Dobb's Journal: Software Tools for the Professional Programmer, 25(11), 120–123.

[crf370055-bib-0005] Bresson, G. , Alsayed, Z. , Yu, L. , & Glaser, S. (2017). Simultaneous localization and mapping: A survey of current trends in autonomous driving. IEEE Transactions on Intelligent Vehicles, 2(3), 194–220.

[crf370055-bib-0006] Burusa, A. K. , van Henten, E. J. , & Kootstra, G. (2022). Attention‐driven active vision for efficient reconstruction of plants and targeted plant parts. *arXiv preprint arXiv:2206.10274*.

[crf370055-bib-0007] Chen, S. , Brahma, S. , Mackay, J. , Cao, C. , & Aliakbarian, B. (2020). The role of smart packaging system in food supply chain. Journal of Food Science, 85(3), 517–525. 32056210 10.1111/1750-3841.15046

[crf370055-bib-0008] Chiu, C. L. , Ho, H.‐C. , Yu, T. , Liu, Y. , & Mo, Y. (2021). Exploring information technology success of augmented reality retail applications in retail food chain. Journal of Retailing and Consumer Services, 61, 102561.

[crf370055-bib-0009] Çöltekin, A. , Lochhead, I. , Madden, M. , Christophe, S. , Devaux, A. , Pettit, C. , Lock, O. , Shukla, S. , Herman, L. , & Stachoň, Z. (2020). Extended reality in spatial sciences: A review of research challenges and future directions. ISPRS International Journal of Geo‐Information, 9(7), 439.

[crf370055-bib-0010] Davis, K. F. , Downs, S. , & Gephart, J. A. (2021). Towards food supply chain resilience to environmental shocks. Nature Food, 2(1), 54–65. 37117650 10.1038/s43016-020-00196-3

[crf370055-bib-0011] Debeunne, C. , & Vivet, D. (2020). A review of visual‐LiDAR fusion based simultaneous localization and mapping. Sensors, 20(7), 2068. 32272649 10.3390/s20072068 PMC7181037

[crf370055-bib-0012] Desai, P. R. , Desai, P. N. , Ajmera, K. D. , & Mehta, K. (2014). A review paper on oculus rift—A virtual reality headset. *arXiv preprint arXiv:1408.1173*.

[crf370055-bib-0013] Dirksen, J. (2013). Learning Three.js: The JavaScript 3D library for WebGL (Vol. 3). Packt Publishing Livery Place.

[crf370055-bib-0014] Dong, X. , Kim, W.‐Y. , Yu, Z. , Oh, J.‐Y. , Ehsani, R. , & Lee, K.‐H. (2024). Improved voxel‐based volume estimation and pruning severity mapping of apple trees during the pruning period. Computers and Electronics in Agriculture, 219, 108834.

[crf370055-bib-0015] Edemetti, F. , Maiale, A. , Carlini, C. , D'Auria, O. , Llorca, J. , & Tulino, A. M. (2022). Vineyard digital twin: Construction and characterization via UAV images–DIWINE proof of concept. In 2022 IEEE 23rd international symposium on a world of wireless, mobile and multimedia networks (WoWMoM) .

[crf370055-bib-0016] Fang, W. , & An, Z. (2020). A scalable wearable AR system for manual order picking based on warehouse floor‐related navigation. The International Journal of Advanced Manufacturing Technology, 109, 2023–2037.

[crf370055-bib-0017] Flamand, T. , Ghoniem, A. , & Maddah, B. (2023). Store‐wide shelf‐space allocation with ripple effects driving traffic. Operations Research, 71(4), 1073–1092.

[crf370055-bib-0018] Galanakis, C. M. (2020). The food systems in the era of the coronavirus (COVID‐19) pandemic crisis. Foods, 9(4), 523. 32331259 10.3390/foods9040523 PMC7230343

[crf370055-bib-0019] Galanakis, C. M. (2023). The “vertigo” of the food sector within the triangle of climate change, the post‐pandemic world, and the Russian‐Ukrainian war. Foods, 12(4), 721. 36832796 10.3390/foods12040721 PMC9956103

[crf370055-bib-0020] Galanakis, C. M. (2024). The future of food. Foods, 13(4), 506. 38397483 10.3390/foods13040506 PMC10887894

[crf370055-bib-0021] Ghazal, T. , & Alzoubi, H. (2021). Modelling supply chain information collaboration empowered with machine learning technique. Intelligent Automation & Soft Computing, 29(3), 243–257.

[crf370055-bib-0022] Gill, S. S. , Xu, M. , Ottaviani, C. , Patros, P. , Bahsoon, R. , Shaghaghi, A. , Golec, M. , Stankovski, V. , Wu, H. , & Abraham, A. (2022). AI for next generation computing: Emerging trends and future directions. Internet of Things, 19, 100514.

[crf370055-bib-0023] Godde, C. M. , Mason‐D'Croz, D. , Mayberry, D. , Thornton, P. K. , & Herrero, M. (2021). Impacts of climate change on the livestock food supply chain; a review of the evidence. Global Food Security, 28, 100488. 33738188 10.1016/j.gfs.2020.100488 PMC7938222

[crf370055-bib-0024] Goldstein, S. C. , & Budiu, M. (2001). Nanofabrics: Spatial computing using molecular electronics. ACM SIGARCH Computer Architecture News, 29(2), 178–191.

[crf370055-bib-0025] Grandi, F. , Prati, E. , Mangia, G. , & Peruzzini, M. (2023). Development of an AR‐based application for training of warehouse operators. In International symposium on industrial engineering and automation .

[crf370055-bib-0026] Herzig, D. , Nakas, C. T. , Stalder, J. , Kosinski, C. , Laesser, C. , Dehais, J. , Jaeggi, R. , Leichtle, A. B. , Dahlweid, F.‐M. , Stettler, C. , & Bally, L. (2020). Volumetric food quantification using computer vision on a depth‐sensing smartphone: Preclinical study. Journal of Medical Internet Research Mhealth and Uhealth, 8(3), e15294. 10.2196/15294 PMC7142738 32209531

[crf370055-bib-0027] Hocking, J. (2022). Unity in action: Multiplatform game development in C. Simon and Schuster.

[crf370055-bib-0028] Hong, G. , Gan, X. , Leonhardt, C. , Zhang, Z. , Seibert, J. , Busch, J. M. , & Bräse, S. (2021). A brief history of OLEDs—Emitter development and industry milestones. Advanced Materials, 33(9), 2005630. 10.1002/adma.202005630 33458866

[crf370055-bib-0029] Hu, Y. (2023). Agricultural management system based on GPS and GIS. In 2023 world conference on communication & computing (WCONF) .

[crf370055-bib-0030] Huang, M. , Ninić, J. , & Zhang, Q. (2021). BIM, machine learning and computer vision techniques in underground construction: Current status and future perspectives. Tunnelling and Underground Space Technology, 108, 103677.

[crf370055-bib-0031] Huang, Y. , Hsiang, E.‐L. , Deng, M.‐Y. , & Wu, S.‐T. (2020). Mini‐LED, micro‐LED and OLED displays: Present status and future perspectives. Light: Science & Applications, 9(1), 105. 10.1038/s41377-020-0341-9 PMC7303200 32577221

[crf370055-bib-0032] Huynh, T. T. , TonThat, L. , & Dao, S. V. (2022). A vision‐based method to estimate volume and mass of fruit/vegetable: Case study of sweet potato. International Journal of Food Properties, 25(1), 717–732.

[crf370055-bib-0033] Jagtap, S. , Saxena, P. , & Salonitis, K. (2021). Food 4.0: Implementation of the augmented reality systems in the food industry. Procedia CIRP, 104, 1137–1142.

[crf370055-bib-0034] Javed, A. R. , Shahzad, F. , ur Rehman, S. , Zikria, Y. B. , Razzak, I. , Jalil, Z. , & Xu, G. (2022). Future smart cities: Requirements, emerging technologies, applications, challenges, and future aspects. Cities, 129, 103794.

[crf370055-bib-0035] Jia, W. , Ren, Y. , Li, B. , Beatrice, B. , Que, J. , Cao, S. , Wu, Z. , Mao, Z. H. , Lo, B. , & Anderson, A. K. (2022). A novel approach to dining bowl reconstruction for image‐based food volume estimation. Sensors, 22(4), 1493. 35214399 10.3390/s22041493 PMC8877095

[crf370055-bib-0036] J'lali, Y. (2020). DirectX 12: Performance comparison between single‐and multithreaded rendering when culling multiple lights. Faculty of Computing, Blekinge Institute of Technology.

[crf370055-bib-0037] Kadam, P. , Pandya, S. , Phansalkar, S. , Sarangdhar, M. , Petkar, N. , Kotecha, K. , & Garg, D. (2022). FVEstimator: A novel food volume estimator Wellness model for calorie measurement and healthy living. Measurement, 198, 111294.

[crf370055-bib-0038] Kalinov, I. , Trinitatova, D. , & Tsetserukou, D. (2021). Warevr: Virtual reality interface for supervision of autonomous robotic system aimed at warehouse stocktaking. In 2021 IEEE international conference on systems, man, and cybernetics (SMC) .

[crf370055-bib-0039] Karada, M. S. , Bajpai, R. , Singh, M. , Singh, A. K. , Agnihotri, D. , & Singh, B. K. (2023). A review on advances in agriculture and agroforestry with GPS and GIS. International Journal of Plant and Soil Science, 35(6), 150–160.

[crf370055-bib-0040] Katkani, D. , Babbar, A. , Mishra, V. K. , Trivedi, A. , Tiwari, S. , & Kumawat, R. K. (2022). A review on applications and utility of remote sensing and geographic information systems in agriculture and natural resource management. International Journal of Environment and Climate Change, 12, 1–18.

[crf370055-bib-0041] Kelemen, D. , & Szénási, S. (2023). Optimization and representation of a network of food delivery drones in simulation. In 2023 IEEE 21st jubilee international symposium on intelligent systems and informatics (SISY) .

[crf370055-bib-0042] Khanal, S. , Kc, K. , Fulton, J. P. , Shearer, S. , & Ozkan, E. (2020). Remote sensing in agriculture—Accomplishments, limitations, and opportunities. Remote Sensing, 12(22), 3783.

[crf370055-bib-0043] Kim, W. B. , & Choo, H. J. (2023). How virtual reality shopping experience enhances consumer creativity: The mediating role of perceptual curiosity. Journal of Business Research, 154, 113378.

[crf370055-bib-0044] Konstantakopoulos, F. S. , Georga, E. I. , & Fotiadis, D. I. (2023). An automated image‐based dietary assessment system for mediterranean foods. IEEE Open Journal of Engineering in Medicine and Biology, 4, 45–54. 37223053 10.1109/OJEMB.2023.3266135 PMC10202193

[crf370055-bib-0045] Kumar, A. , Ahamad, S. , Kumar, M. , Bihari, C. , Singh, S. , Pandey, V. , Alam, K. , Singh, S. , & Gautam, P. (2022). An innovative drones technology in agriculture: A review. The Pharma Innovation Journal, 11(12), 279–286.

[crf370055-bib-0046] Lapinski, P. (2017). Vulkan cookbook. Packt Publishing Ltd.

[crf370055-bib-0047] Lillford, P. , & Hermansson, A.‐M. (2021). Global missions and the critical needs of food science and technology. Trends in Food Science & Technology, 111, 800–811.

[crf370055-bib-0048] Lin, C.‐C. , Peng, Y.‐C. , & Kang, J.‐R. (2024). Joint green dynamic order batching and picker routing problem using PSO with global worst experience. Applied Soft Computing, 154, 111336.

[crf370055-bib-0049] Linowes, J. , & Babilinski, K. (2017). Augmented reality for developers: Build practical augmented reality applications with unity, ARCore, ARKit, and Vuforia. Packt Publishing Ltd.

[crf370055-bib-0050] Lo, F. P. W. , Sun, Y. , Qiu, J. , & Lo, B. (2020). Image‐based food classification and volume estimation for dietary assessment: A review. IEEE Journal of Biomedical and Health Informatics, 24(7), 1926–1939. 10.1109/jbhi.2020.2987943 32365038

[crf370055-bib-0051] Lu, J. , Fu, H. , Tang, X. , Liu, Z. , Huang, J. , Zou, W. , Chen, H. , Sun, Y. , Ning, X. , Li, J. , & Li, J. (2024). GOA‐optimized deep learning for soybean yield estimation using multi‐source remote sensing data. Scientific Reports, 14(1), 7097. 38528045 10.1038/s41598-024-57278-6 PMC10963745

[crf370055-bib-0052] Lu, Y. , Stathopoulou, T. , Vasiloglou, M. F. , Pinault, L. F. , Kiley, C. , Spanakis, E. K. , & Mougiakakou, S. (2020). goFOODTM: An artificial intelligence system for dietary assessment. Sensors, 20(15), 4283. 32752007 10.3390/s20154283 PMC7436102

[crf370055-bib-0053] Ma, P. , Lau, C. P. , Yu, N. , Li, A. , & Sheng, J. (2022). Application of deep learning for image‐based Chinese market food nutrients estimation. Food Chemistry, 373, 130994. 34731793 10.1016/j.foodchem.2021.130994

[crf370055-bib-0054] Macenski, S. , Foote, T. , Gerkey, B. , Lalancette, C. , & Woodall, W. (2022). Robot operating system 2: Design, architecture, and uses in the wild. Science Robotics, 7(66), eabm6074. 35544605 10.1126/scirobotics.abm6074

[crf370055-bib-0055] Macoir, N. , Bauwens, J. , Jooris, B. , Van Herbruggen, B. , Rossey, J. , Hoebeke, J. , & De Poorter, E. (2019). Uwb localization with battery‐powered wireless backbone for drone‐based inventory management. Sensors, 19(3), 467. 30678128 10.3390/s19030467 PMC6386853

[crf370055-bib-0056] Moshood, T. D. , Nawanir, G. , Sorooshian, S. , & Okfalisa, O. (2021). Digital twins driven supply chain visibility within logistics: A new paradigm for future logistics. Applied System Innovation, 4(2), 29.

[crf370055-bib-0100] Nagendra, A. (2019). 5G and its impact on Supply Chain. Management Vision, *77*.

[crf370055-bib-0057] Nagarajan, S. M. , Deverajan, G. G. , Chatterjee, P. , Alnumay, W. , & Muthukumaran, V. (2022). Integration of IoT based routing process for food supply chain management in sustainable smart cities. Sustainable Cities and Society, 76, 103448.

[crf370055-bib-0058] Nasirahmadi, A. , & Hensel, O. (2022). Toward the next generation of digitalization in agriculture based on digital twin paradigm. Sensors, 22(2), 498. 35062459 10.3390/s22020498 PMC8780442

[crf370055-bib-0059] Ndjuluwa, L. N. , Adebisi, J. A. , & Dayoub, M. (2023). Internet of things for crop farming: A review of technologies and applications. Commodities, 2(4), 367–381.

[crf370055-bib-0060] Omia, E. , Bae, H. , Park, E. , Kim, M. S. , Baek, I. , Kabenge, I. , & Cho, B.‐K. (2023). Remote sensing in field crop monitoring: A comprehensive review of sensor systems, data analyses and recent advances. Remote Sensing, 15(2), 354.

[crf370055-bib-0061] Onwude, D. I. , Chen, G. , Eke‐Emezie, N. , Kabutey, A. , Khaled, A. Y. , & Sturm, B. (2020). Recent advances in reducing food losses in the supply chain of fresh agricultural produce. Processes, 8(11), 1431.

[crf370055-bib-0062] Oufqir, Z. , El Abderrahmani, A. , & Satori, K. (2020). ARKit and ARCore in serve to augmented reality. In 2020 international conference on intelligent systems and computer vision (ISCV) .

[crf370055-bib-0063] Paszke, A. , Gross, S. , Massa, F. , Lerer, A. , Bradbury, J. , Chanan, G. , Killeen, T. , Lin, Z. , Gimelshein, N. , & Antiga, L. (2019). Pytorch: An imperative style, high‐performance deep learning library. In Wallach, H. , Larochelle, H. , Beygelzimer, A. , d'Alché‐Buc, F. , Fox, E. & Garnett, R. (Eds.), Advances in neural information processing systems (Vol. 32, pp. 8024–8035). Neural Information Processing Systems Foundation Inc. (NeurIPS).

[crf370055-bib-0064] Peladarinos, N. , Piromalis, D. , Cheimaras, V. , Tserepas, E. , Munteanu, R. A. , & Papageorgas, P. (2023). Enhancing smart agriculture by implementing digital twins: A comprehensive review. Sensors, 23(16), 7128. 37631663 10.3390/s23167128 PMC10459062

[crf370055-bib-0065] Poppiel, R. R. , Lacerda, M. P. , Safanelli, J. L. , Rizzo, R. , Oliveira, M. P. Jr , Novais, J. J. , & Demattê, J. A. (2019). Mapping at 30 m resolution of soil attributes at multiple depths in midwest Brazil. Remote Sensing, 11(24), 2905.

[crf370055-bib-0066] Purcell, W. , Neubauer, T. , & Mallinger, K. (2023). Digital twins in agriculture: Challenges and opportunities for environmental sustainability. Current Opinion in Environmental Sustainability, 61, 101252.

[crf370055-bib-0067] Pylianidis, C. , Osinga, S. , & Athanasiadis, I. N. (2021). Introducing digital twins to agriculture. Computers and Electronics in Agriculture, 184, 105942.

[crf370055-bib-0068] Rusu, R. B. , & Cousins, S. (2011). 3D is here: Point Cloud Library (PCL). In 2011 IEEE international conference on robotics and automation .

[crf370055-bib-0069] Sadeeq, M. M. , Abdulkareem, N. M. , Zeebaree, S. R. , Ahmed, D. M. , Sami, A. S. , & Zebari, R. R. (2021). IoT and Cloud computing issues, challenges and opportunities: A review. Qubahan Academic Journal, 1(2), 1–7.

[crf370055-bib-0070] Sari, Y. A. , & Gofuku, A. (2023). Measuring food volume from RGB‐depth image with point cloud conversion method using geometrical approach and robust ellipsoid fitting algorithm. Journal of Food Engineering, 358, 111656.

[crf370055-bib-0071] Shreiner, D. (2009). OpenGL programming guide: The official guide to learning OpenGL, versions 3.0 and 3.1. Pearson Education.

[crf370055-bib-0072] Shurlaeva, E. , Tokarev, K. , & Sanzhapov, B. K. (2021). Satellite monitoring and visualization of vegetation indices for assessing crop productivity. Journal of Physics: Conference Series, 2060(1), 012018.

[crf370055-bib-0073] Simonetti Ibañez, A. , & Paredes Figueras, J. (2013). Vuforia v1. 5 SDK: Analysis and evaluation of capabilities. Universitat Politècnica de Catalunya.

[crf370055-bib-0074] Smith, S. P. , Adam, M. T. , Manning, G. , Burrows, T. , Collins, C. , & Rollo, M. E. (2022). Food volume estimation by integrating 3D image projection and manual wire mesh transformations. IEEE Access, 10, 48367–48378.

[crf370055-bib-0075] Soon, P. S. , Lim, W. M. , & Gaur, S. S. (2023). The role of emotions in augmented reality. Psychology & Marketing, 40(11), 2387–2412.

[crf370055-bib-0076] Sugimoto, K. , Ishihara, S. , & Itoh, M. (2024). Mobile robot navigation in warehouses by MPC handling multiple travel strategies considering independent safety LiDAR. In 2024 IEEE/SICE international symposium on system integration (SII) .

[crf370055-bib-0077] Sun, M. , Lu, L. , Ni, H. , Wang, Y. , & Gao, J. (2022). Research on dynamic path planning method of moving single target based on visual AGV. SN Applied Sciences, 4(3), 86.

[crf370055-bib-0078] Tagliavini, G. , Defraeye, T. , & Carmeliet, J. (2019). Multiphysics modeling of convective cooling of non‐spherical, multi‐material fruit to unveil its quality evolution throughout the cold chain. Food and Bioproducts Processing, 117, 310–320.

[crf370055-bib-0079] Thirisha, R. , Sugumar, D. , Sugitha, K. , & Jose, A. V. (2023). Precision agriculture: IoT based system for real‐time monitoring of paddy growth. In 2023 international conference on sustainable emerging innovations in engineering and technology (ICSEIET) .

[crf370055-bib-0080] Usukura, N. , Minoura, K. , & Maruyama, R. (2023). Novel pancake‐based HMD optics to improve light efficiency. Journal of the Society for Information Display, 31(5), 344–354.

[crf370055-bib-0081] Venter, H. , & Ogterop, W. (2022). Unreal engine 5 character creation, animation, and cinematics: Create custom 3D assets and bring them to life in unreal engine 5 using MetaHuman, Lumen, and Nanite. Packt Publishing Ltd.

[crf370055-bib-0082] Verdouw, C. , Tekinerdogan, B. , Beulens, A. , & Wolfert, S. (2021). Digital twins in smart farming. Agricultural Systems, 189, 103046.

[crf370055-bib-0083] Wang, W. , Min, W. , Li, T. , Dong, X. , Li, H. , & Jiang, S. (2022). A review on vision‐based analysis for automatic dietary assessment. Trends in Food Science & Technology, 122, 223–237.

[crf370055-bib-0084] Wang, W. , Wang, F. , Song, W. , & Su, S. (2020). Application of augmented reality (AR) technologies in inhouse logistics. In E3S web of conferences .

[crf370055-bib-0085] Wang, Y. , Yuan, Y. , Yuan, F. , Ata‐UI‐Karim, S. T. , Liu, X. , Tian, Y. , Zhu, Y. , Cao, W. , & Cao, Q. (2023). Evaluation of variable application rate of fertilizers based on site‐specific management zones for winter wheat in small‐scale farming. Agronomy, 13(11), 2812.

[crf370055-bib-0086] White, G. , Zink, A. , Codecá, L. , & Clarke, S. (2021). A digital twin smart city for citizen feedback. Cities, 110, 103064.

[crf370055-bib-0087] Wu, W. , Zhao, Z. , Shen, L. , Kong, X. T. , Guo, D. , Zhong, R. Y. , & Huang, G. Q. (2022). Just trolley: Implementation of industrial IoT and digital twin‐enabled spatial‐temporal traceability and visibility for finished goods logistics. Advanced Engineering Informatics, 52, 101571.

[crf370055-bib-0088] Xu, Z. , Qi, M. , Wu, Y. , Hao, X. , & Yang, Y. (2021). City information modeling: State of the art. Applied Sciences, 11(19), 9333.

[crf370055-bib-0089] Xu, Z. , Zheng, N. , Lv, Y. , Fang, Y. , & Vu, H. L. (2024). Analyzing scenario criticality and rider's intervention behavior during high‐level autonomous driving: A VR‐enabled approach and empirical insights. Transportation Research Part C: Emerging Technologies, 158, 104451.

[crf370055-bib-0090] Yigitbas, E. , Jovanovikj, I. , Scholand, J. , & Engels, G. (2020). VR training for warehouse management. In Proceedings of the 26th ACM symposium on virtual reality software and technology .

[crf370055-bib-0091] Yousefi, M. R. , & Razdari, A. M. (2015). Application of GIS and GPS in precision agriculture (a review). International Journal of Advanced Biological and Biomedical Research, 3(1), 7–9.

[crf370055-bib-0092] Yuan, C.‐D. , Chen, J.‐K. , Wang, F. , Ouyang, J.‐J. , Jing, T. , Wang, X.‐F. , Yang, B. , & Shao, Z.‐G. (2023). Research on 3D reconstruction method and application of food in stroke patients based on RGB‐D image. Journal of Mechanics in Medicine and Biology, 23(02), 2350026.

[crf370055-bib-0093] Zawadzki, P. , Żywicki, K. , Buń, P. , & Górski, F. (2020). Employee training in an intelligent factory using virtual reality. IEEE Access, 8, 135110–135117.

[crf370055-bib-0094] Zhang, J. , Li, Y. , & Lu, Z. (2024). Multi‐period vehicle routing problem with time windows for drug distribution in the epidemic situation. Transportation Research Part C: Emerging Technologies, 160, 104484.

[crf370055-bib-0095] Zhang, L. , & Zhang, L. (2022). Artificial intelligence for remote sensing data analysis: A review of challenges and opportunities. IEEE Geoscience and Remote Sensing Magazine, 10(2), 270–294.

[crf370055-bib-0096] Zhang, S. , Callaghan, V. , & Che, Y. (2023). Image‐based methods for dietary assessment: A survey. Journal of Food Measurement and Characterization, 18(3), 1–17.

[crf370055-bib-0097] Zheng, X. , Liu, C. , Gong, Y. , Yin, Q. , Jia, W. , & Sun, M. (2023). Food volume estimation by multi‐layer superpixel. Mathematical Biosciences and Engineering, 20(4), 6294–6311. 37161107 10.3934/mbe.2023271

[crf370055-bib-0098] Zuckerman, N. , Cohen, Y. , Alchanatis, V. , & Lensky, I. M. (2024). Toward precision agriculture in outdoor vertical greenery systems (VGS): Monitoring and early detection of stress events. Remote Sensing, 16(2), 302.

